# Supplementary material for: Estimating the total number of phosphoproteins and phosphorylation sites in eukaryotic proteomes
Source: Gigascience. 2017 Jan 7;6(2):1–11. doi: 10.1093/gigascience/giw015 (PMC5466708; doi:10.1093/gigascience/giw015)
Supplement: GIGA-D-16-00079_Revision_1.pdf [file giw015_giga-d-16-00079_revision_1.pdf]

Manuscript

Click here to download Manuscript Vlastaridis_et_al_2016_gigascience_R1 for production.docx

Title: Estimating the total number of phosphoproteins and phosphorylation sites in eukaryotic proteomes.

Panayotis Vlastaridis[1], Pelagia Kyriakidou[1], Anargyros Chaliotis[1], Yves Van de Peer[2,3,4] Stephen G. Oliver[5], Grigoris D. Amoutzias[1]*

*correspondence should be addressed to: amoutzias@bio.uth.gr

1 Bioinformatics Laboratory, Department of Biochemistry and Biotechnology, University of Thessaly, Larisa, 41500, Greece

2 Department of Plant Systems Biology, VIB and Department of Plant Biotechnology and Bioinformatics, Ghent University, B-9052 Ghent, Belgium

3 Bioinformatics Institute Ghent, Technologiepark 927, B-9052 Ghent, Belgium

4 Department of Genetics, Genomics Research Institute, University of Pretoria, Pretoria 0028, South Africa

5 Cambridge Systems Biology Centre & Department of Biochemistry, University of Cambridge, Cambridge CB2 1GA, UK.

Abstract

Background: Phosphorylation is the most frequent post-translational modification made to proteins and may regulate protein activity as either a molecular digital switch or a rheostat. Despite the cornucopia of high-throughput (HTP) phosphoproteomic data in the last decade, it remains unclear how many proteins are phosphorylated and how many phosphorylation sites (p-sites) can exist in

total within a eukaryotic proteome. We present the first reliable estimates of the total number of phosphoproteins and phosphorylation sites (p-sites), for four eukaryotes (human, mouse, *Arabidopsis,* and yeast).

Results: In all, 187 HTP phosphoproteomic datasets were filtered, compiled and studied along with two low-throughput (LTP) compendia. Estimates of the number of phosphoproteins and p-sites were inferred by two methods: Capture-Recapture, and fitting the saturation curve of cumulative redundant vs. cumulative non-redundant phosphoproteins/p-sites. Estimates were also adjusted for different levels of noise within the individual datasets and other confounding factors. We estimate that in total, 13,000, 11,000 and 3,000 phosphoproteins and 230,000, 156,000 and 40,000 p-sites exist in human, mouse and yeast, respectively, whereas estimates for Arabidopsis were not as reliable.

Conclusions: Most of the phosphoproteins have been discovered for human, mouse and yeast, while the dataset for *Arabidopsis* is still far from complete. The datasets for p-sites are not as close to saturation as those for phosphoproteins, Integration of the LTP data suggests that current HTP phosphoproteomics appears to be capable of capturing 70-95% of total phosphoproteins, but only 40-60% of total p-sites.

Keywords: Capture-Recapture /Curve-Fitting /Phosphoproteomics /total number of phosphoproteins /total number of phosphorylation sites/yeast/human/mouse/Arabidopsis.

Background

Phosphorylation is the most frequent post-translational modification made to proteins [1] and may regulate protein activity as either a molecular digital switch or a rheostat. Enzyme activity, complex

formation, subcellular localization, or degradation are some of the functions that may be regulated via allosteric or orthosteric effects [2]. Phosphorylation/dephosphorylation is also a key component of signal transduction. More than one switch of this kind may be present in a protein and phosphorylation events may be independent of each other, or there may be interdependencies between them or even with other types of switches [3]. In addition, phosphorylation affects the evolution of a genome [4].

It is of paramount importance to know which proteins are phosphorylated and on which of their amino acids. In spite of all this, it remains unclear how many proteins are phosphorylated and how many phosphorylation sites (p-sites) can exist within a proteome. This question will be answered when all of them have been identified and novel ones are no longer discovered. Until that point has been reached, however, it is necessary to have a reasonable estimate of their total numbers. Such an estimate will permit us to determine the limits of our current knowledge and will allow us to appreciate how much still remains to be discovered. It will also provide a critical evaluation of the efficacy of current approaches and indicate what novel strategies and technologies will need to be developed in order to achieve the ultimate goal of obtaining a comprehensive inventory of all phosphoproteins, their p-sites and the physiological and developmental contexts in which they are modified.

At present, values for these numbers remain in the realm of speculation. It has been suggested that the biological activity of between 1/3 and 2/3 of an organism's proteome could be regulated by protein phosphorylation [5–8]. In the specific case of the human proteome, it has been proposed that 57,000, 500,000, 700,000, or even 1,000,000 p-sites may exist [9–12]. Sharma *et al.* performed a

deep phosphoproteome analysis on HeLa cells and estimated that at least 75% of the proteome expressed in those cells can be phosphorylated, and this number may well rise to 90%, if phosphoproteomic experiments are performed at higher coverage [13]. In an effort to provide a reasonable and statistically defensible estimate based on current knowledge, we have mined over 1000 articles from the literature and gathered and filtered 187 publicly available HTP phosphoproteomic datasets from four well-studied species. By implementing two independent statistical methods - the Capture-Recapture method, and Curve-Fitting on the saturation curve of redundant phosphoproteins/p-sites vs non-redundant phosphoproteins/p-sites - we have obtained, for the first time, a reliable estimate of their total number for humans and three other model eukaryotes.

Data Description

Over 1000 relevant articles were retrieved from PubMed with the keywords “phosphoproteomic OR phosphoproteomics” and were manually inspected for available raw data in human, mouse, *Arabidopsis,* and yeast. Only articles that provided the sequences of phosphopeptides and the exact p-site location with algorithm-specific confidence scores were retained. These phosphopeptides were further filtered with a cut-off criterion of 99% correct phosphopeptide sequence identification and 99% correct p-site localization, to ensure that only data of very high quality were used in the subsequent analyses. Finally, phosphopeptides that exactly matched two or more genes/proteins were removed. Thus, 97, 42, 28 and 20 HTP datasets were retained for human, mouse, *Arabidopsis*, and budding yeast respectively (see supplementary files S1-S5). The human and mouse proteomes were retrieved from ENSEMBL VEGA, based on the GRCh38 and GRCm38 reference assemblies respectively (December 2015) [14]. For every protein-encoding gene that was annotated by VEGA, only the longest peptide was retained. The *Arabidopsis* proteome was retrieved from TAIR 10 [15],

whereas the budding yeast proteome was derived from the *Saccharomyces* Genome Database (SGD) [16].

Human and mouse p-sites that were identified by LTP technologies are considered to be of higher quality/confidence and were retrieved from the Phosphosite plus database [17]. The downloaded phospho-motifs were mapped to the Ensembl peptide sequences. Only p-sites whose coordinates matched exactly between the Swissprot (provided by Phosphosite-plus) and Ensembl proteins were retained. Yeast LTP p-sites were retrieved from the PhosphoGrid2 database [8,18]. No LTP compendium was available for *Arabidopsis*.

Analyses

Estimation of the total number of yeast phosphoproteins and p-sites

S. cerevisiae (budding yeast) is the best-studied unicellular eukaryote and harbours only ~6,000 proteins [19,20]. Twenty HTP phosphoproteomic datasets from 18 articles have been collected from this organism, under a reasonably wide range of conditions, and more than 70% of its entire proteome is detectable by MS/MS technology in a single experiment [21,22]. In addition, a very comprehensive compendium of LTP, but high quality, p-sites has been compiled by the PhosphoGrid2 database [8]. Therefore, yeast is the ideal system with which to estimate the total number of phosphoproteins and p-sites. For these reasons, we will describe the complete process of the analyses performed on the yeast proteome to illustrate our approach. We will then summarize the outcomes of similar analyses performed with the proteomes of the other three species examined.

To date, 2,587 phosphoproteins and 13,244 p-sites (2,633 and 14,341 including the PhosphoGrid LTP data; see supplementary file S1) have been discovered, probably with some of them being false-positives. The saturation level of the yeast phosphoproteins (based on the HTP data) is depicted in Figure 1A whereas the estimates of their total number, based on different methods and data treatments is depicted in Figure 1B. It is evident, especially from Figure 1A, that the detection of phosphoproteins with HTP methods has approached saturation. Assuming 1% noise in each experiment, the Curve-Fitting method estimates ~2,400 true-positive phosphoproteins, whereas the Capture-Recapture method estimates ~2,800. In addition, curve-fitting estimates based on highly confident phosphoproteins that have been detected in three or more experiments (this criterion is based on a previous analysis [5] - designated as 3X) suggests a total of ~2,300 phosphoproteins. Therefore a gross estimate of 2,300-2,800 phosphoproteins, ~40-50% of the proteome, seems as a reasonable one, based solely on the current HTP technologies. These conclusions appear robust, even if the order of the largest experiment is perturbed (as first –best_start in graph; or last –best_end in graphs; in the series) and even if only half of the experiments are used in Curve-Fitting (see Figure 1B). Interestingly, Beltrao *et al.*, also suggested that HTP phosphoproteomic studies have revealed about 80-90% of all *S. cerevisiae* phosphoproteins [23].

Concerning the saturation level of p-sites, it is evident, especially from Figure 1C, that their detection is approaching saturation, although this trend is less marked than it is for the total number of phosphoproteins. Assuming 1% noise in each experiment, the Curve-Fitting method estimates ~15,000 true positive p-sites, whereas the Capture-Recapture method raises this estimate to ~21,000.

Curve-Fitting based on highly confident p-sites that have been detected in 3 or more experiments, failed to provide a reasonable estimate.

The above estimates are based solely on 20 HTP experiments. Nevertheless, several experimental and computational studies have reported that HTP phosphoproteomic experiments may fail to capture many known p-sites, depending on various parameters and protocols [5,18,24–28]. To control for this factor, the LTP (high confidence) data from PhosphoGrid2 were employed as well and were merged into one non-redundant LTP dataset. Similarly, all HTP experiments were merged into one non-redundant HTP dataset. Next, the Capture-Recapture method was implemented by using as input two datasets, the merged HTP one and the PhosphoGrid2 LTP one. This time, the estimate significantly increased from 21,000 to 40,000 p-sites. On the contrary, the equivalent analysis estimated 2,951 total phosphoproteins, which is very close to the one generated by the Capture-Recapture method (2,772) that used the 15 largest HTP datasets individually. We believe that the analysis incorporating the LTP data provides a more realistic total estimate than an analysis based solely on HTP data. Consequently, the current HTP technologies have the potential to capture the vast majority (94%) of the yeast phosphoproteome, but only ~ 53% of the total p-sites.

Similar analyses to those performed on the *S. cerevisiae* proteome were also executed with three other species. The results are presented in Figures 2 (*Homo sapiens*), 3 (*Mus musculus*), and 4 (*Arabidopisis thaliana*), and Table 1 compares the outcomes of the analyses of all four proteomes. In the Table, the most reliable estimates, obtained by incorporating both the HTP and LTP non-redundant datasets are highlighted in bold.

Table 1. **Estimates on the total number of phosphoproteins and p-sites for the various species, based on different analyses.**

		Human	Mouse	Arabidopsis	Yeast
PROTEINS	current	10456	6512	4930	2587
	current_3X	6683	3827	1815	1630
	**Rcapture_HTP_vs_LTP**	**12844**	**11190**	**NA**	**2951**
	Rcapture_1%_noise	10239	8346	6531	2772
	CF_1%_noise	9160	7213	4292	2373
	CF_3X	7582	6789	NA	2297
	CF_best_start_1%_noise	8803	7167	4558	2328
	CF_best_end_1%_noise	8775	7099	4292	2304
	CF_half_exp_1%_noise	7885	6329	2373	2257
P-SITES	current	86181	36438	14796	13244
	current_3X	27110	10384	3078	4156
	**Rcapture_HTP_vs_LTP**	**229616**	**155668**	**NA**	**40350**
	Rcapture_1%_noise	124985	71456	27815	21343
	CF_1%_noise	94670	54031	23531	14533
	CF_3X	91500	NA	34457	NA
	CF_best_start_1%_noise	82092	45797	15122	12962
	CF_best_end_1%_noise	86723	49122	23531	14496
	CF_half_exp_1%_noise	89639	36615	6016	11980

Second column denotes the analysis and datasets: current: experimentally identified; current_3X: experimentally identified in three or more experiments; Rcapture_HTP_vs_LTP: The Capture-Recapture analysis that used the HTP compendium and the LTP compendium (shown in **bold** as the most reliable estimate); Rcapture_1%_noise: The Capture-Recapture analysis assuming 1% noise in each dataset; CF_1%_noise: The Curve-Fitting analysis assuming 1% noise; CF_3X: The Curve-Fitting analysis based on the datasets that have been identified in three or more experiments. CF_best_start_1%_noise: The Curve-Fitting analysis assuming 1% noise and changing the

order of the largest experiment as first; CF_best_end_1%_noise: The Curve-Fitting analysis assuming 1% noise and changing the order of the largest experiment as last; CF_half_exp_1%_noise: The Curve-Fitting analysis assuming 1% noise and using only the first half of experiments.

Estimation of the total number of phosphoproteins and p-sites in the two mammalian proteomes

As expected, the organism with the most data is *Homo sapiens*, where 97 HTP experimental datasets have so far generated 86,181 p-sites in 10,456 phosphoproteins (see supplementary file S2), using the same filtering criteria as yeast. Mouse is a mammal that is used extensively as a model for understanding human biology and is a rather close evolutionary relative of our own species with a time divergence of about 90 million years. Budding yeast, by comparison, is a unicellular fungus that diverged from its most-recent common ancestor with humans ~1.3 billion years ago [29]. In addition, human and mouse have a very similar number of protein-coding genes, ~20,000 [30,31]. Therefore, estimates on the mouse phosphoproteome and p-sites are expected to be of the same magnitude as those for human, thus serving as a quality control for our estimates for humans, above. Nevertheless, the number of publicly available datasets for mouse is not as high, with 42 HTP experimental datasets that generated (with our stringent filtering criteria) 36,438 detected p-sites in 6,512 phosphoproteins so far (see supplementary file S3). Of note, in our analyses, the VEGA annotated mouse proteome was used; this consists of ~16,000 protein-coding genes, although the total number is estimated at ~20,000. Therefore, all mouse estimates obtained in this analysis have

been adjusted upwards by 25%, in order to make reasonable estimates for the complete mouse proteome and not for the VEGA highly annotated subset.

It is evident, especially from Figure 2A, that the detection of phosphoproteins (based on HTP data) in the human proteome has approached saturation, while that for mouse phosphoproteins (Figure 3A) has yet to plateau. Based on the HTP data alone, the Capture-Recapture method estimates 10,200 true-positive phosphoproteins for humans, compared to ~8,300 for mouse (both with an assumed error rate of 1%). A jackknife analysis on the Capture-Recapture method suggested 8,500 ± 960 and 8,600 ± 530 phosphoproteins as the lower bound for human and mouse, respectively. It should be noted that the jackknife analyses do not use the largest 15 datasets, but randomly selected ones.

The Curve-Fitting method (with 1% error rate, whenever applicable) on the HTP data and their various perturbations (order of largest experiment, using half the datasets, using phosphoproteins detected in 3 or more experiments) estimates 7,600 - 9,200 and 6,300 - 7,200 phosphoproteins for human and mouse respectively. From these combined analyses of Capture-Recapture and Curve-Fitting on HTP data alone, gross estimates of 7,600 – 10,200 phosphoproteins in humans, and 6,300 – 8,300 in mouse, appears reasonable. It is reassuring that the estimates for the mouse phosphoproteome are not so different to those for the human despite the fact that there are ~50% fewer datasets for mouse.

Concerning the saturation level of p-sites, Figures 2C and 3C suggest that their detection (based on HTP data alone) in human and mouse is approaching saturation, but less rapidly than are the phosphoprotein data (a similar disparity was observed with yeast, above). The Capture-Recapture

method on the HTP data estimates 125,000 p-sites for human and 71,000 for mouse. A jackknife analysis on the Capture-Recapture method suggested 69,000 ± 18,000 and 46,000±8,000 p-sites as the lower bound for human and mouse, respectively.

The Curve-Fitting method (assuming 1% error rate) on the HTP data and their various perturbations (order of largest experiment, using half the datasets, using p-sites detected in 3 or more experiments) estimate 82,000-95,000 and 37,000-54,000 p-sites for human and mouse respectively. Of note, no reasonable estimate was obtained for mouse when using p-sites detected in 3 or more experiments.

The above estimates are based solely on 97 (human) and 42 (mouse) HTP experiments. To control for the fact that HTP technologies may not be able to detect the whole phosphoproteome, a compendium of LTP phosphoproteins/p-sites from Phosphosite plus was used. In addition, all HTP experiments were merged into one non-redundant HTP dataset for each species separately. This time, the Capture-Recapture method was implemented in each species separately by using, as input, two datasets (instead of 15 individual ones as before), the merged HTP one and the Phosphosite LTP one. Notably, the maximum estimate of total p-sites significantly increased from 125,000 to 230,000 for human and from 71,000 to 156,000 for mouse. In contrast, the equivalent increase of maximum estimate for phosphoproteins was from 10,200 to 12,800 for human and from 8,300 to 11,200 for mouse. A reasonable interpretation is that the Capture-Recapture estimates that employ the LTP data are more realistic and that the current HTP technologies alone have the potential to capture the majority of the human (80%) and mouse (74%) phosphoproteome, but only 54% and 46% of their total p-sites. The estimates of the number of mouse phosphoproteins and p-sites are about 13% and

32% lower than those of the human phosphoproteins and p-sites respectively (See Table 1 for details).

Estimation of *Arabidopsis* phosphoproteins and p-sites

Arabidopsis thaliana is a model flowering plant (a eu-dicot) with ~28,000 protein-encoding genes [15] and multiple tissues and cell-types. By mining the literature and applying our stringent criteria, we have collected 28 HTP experimental datasets that generated 14,796 p-sites in 4,930 phosphoproteins (see supplementary file S4). The saturation level of the *Arabidopsis* phosphoproteins is depicted in Figure 4A, while the estimates on their total number, based on the different methods and data treatments, are depicted in Figure 4B. It is evident, especially from Figure 4A (see final data point), that the detection of phosphoproteins is far from approaching saturation. Notably, the last experiment detected a lot of new phosphoproteins, thus casting even more doubt as to whether there are sufficient data to provide any reliable estimates. Curve-Fitting estimates based on highly confident phosphoproteins that have been detected in 3 or more experiments failed to provide a reasonable estimate. To make matters worse, Curve-Fitting based on half of the experiments provided an unrealistically low number. Considering all the above major concerns, an estimate of 4,300 phosphoproteins provided by Curve-Fitting seems unrealistic. On the contrary, the Capture-Recapture method provided an estimate of 6,500 phosphoproteins, but considering the significant contribution of the last experiment, this estimate should be interpreted as a very conservative lower-bound. Apparently, the publicly available data have not yet reached saturation and thus are not sufficient to provide a reliable estimate of the total number of

phosphoproteins. As a consequence, any attempt to estimate the total number of p-sites in *Arabidopsis* is even more problematic.

Concerning the saturation level of p-sites, it is evident from Figure 4C (see final data point) that their detection is also far from approaching saturation. Curve-fitting provided a dubious estimate of ~24,000 p-sites. In addition, curve-fitting estimates based on highly confident p-sites that have been detected in 3 or more experiments provide an estimate of ~ 35,000 p-sites, but a visual inspection of the curve suggests that it still follows a linear mode and therefore this estimate is, to say the least, dubious. The Capture-Recapture method estimates ~28,000 total p-sites. Therefore, a gross estimate of 24,000-35,000 p-sites is currently suggested by the data, but should be considered of very low confidence.

Discussion.

Literature mining and stringent filtering of 187 publicly available HTP phosphoproteomic datasets was performed in this study so as to compile the most comprehensive data compendia for human and three model eukaryotes: mouse, *Arabidopsis* and yeast. Two publicly available database compendia of low-throughput, high-quality data (from PhosphoGrid2 and Phosphosite plus), which serve as “gold-standards” were also integrated. Based on these compendia, estimates of the total number of phosphoproteins and p-sites within each proteome were calculated using two different methods: i) the Capture-Recapture approach that is widely used in ecology and epidemiology to estimate population size, ii) parameter optimization (Curve-Fitting) on the saturation curve of cumulative redundant vs cumulative non-redundant phosphoproteins/p-sites. Estimates for both methods were

also re-adjusted for various levels of noise and perturbations within the individual data. This analysis has generated what we believe is the first set of approximate estimates of the total number of phosphoproteins/p-sites in a range of species that is based on established computational and statistical approaches and which also critically assesses their validity.

The field of phosphoproteomics still faces significant experimental and computational challenges [32]. Several studies have reported that HTP phosphoproteomic experiments alone may fail to capture many known p-sites, depending on various parameters and protocols [5,18,24–28]. For example, consecutive proteolytic digestion by two or more enzymes increased phosphoprotein and p-site detection by 40-70%, compared to an experiment that used only one enzyme [25,26,28]. Along the same line of evidence, a previous Proteomics analysis on yeast showed that the use of additional proteases, apart from the standard Trypsin resulted in a significant increase of proteomics coverage from 21% to 35% of total Serines, Threonines, Tyrosines [33]. Furthermore, the combined use of LysargiNase with ETD not only increased the phosphoproteome coverage, but also generated spectra that allowed for easier localization of p-sites [34]. Thus, the proteomics community is exploring the consecutive use of many more than one proteolytic enzymes [35]. P-sites are not evenly distributed across the proteome but tend to cluster, especially at disordered regions [5,36–38], thus increasing the probability of missing many neighboring p-sites due to problematic enzymatic digestion of that peptide region. Also, the vast majority of the phosphoproteomic datasets are generated by three enrichment methods (IMAC, TiO_2 and p-Tyr pull down) that are well known to exhibit relatively low overlap among them, due to inherent biases towards certain classes of phosphopeptides [39,40]. Therefore, it is conceivable that a significant fraction of phosphopeptides are still undetectable from the current HTP protocols. Furthermore, several replicates may be needed

to capture a certain phosphoproteome in a certain condition, as revealed by the saturation analysis of four technical replicates of the Tyrosine phosphoproteome in human embryonic stem cells [9]. In addition, our analysis filtered and retained phosphopeptides with very high (≥ 99%) p-site localization probabilities. Therefore, some of the estimates only reflect what the current technologies, under certain filtering criteria, are capable of detecting if many experiments are performed.

Since current HTP phosphoproteomic technologies are unable to capture all known p-sites, a specific Capture-Recapture analysis was performed in yeast, human and mouse separately, where the total number of non-redundant HTP phosphoproteins/p-sites were merged as one experiment and the total number of non-redundant LTP phosphoproteins/p-sites (obtained from PhosphoGrid2 and Phosphosite plus) were merged as a second experiment. In this case, the estimates for phosphoproteins did not change significantly. For yeast, the maximum estimates changed from ~2,800 to 2,950 phosphoproteins (potential HTP detection at 94%); for human, the maximum estimates changed from 10,200 to 12,800 phosphoproteins (potential HTP detection at 80%); for mouse, the maximum estimates changed from 8,300 to 11,200 (potential HTP detection at 74%). Indeed, many lines of evidence suggest that detection of the phosphoproteome for yeast, and humans has approached saturation, but this is less so for mouse. Nevertheless, concerning the total number of p-sites, this particular approach revealed that the current HTP technologies alone are capable of detecting only ~46 – 54% of the total. In yeast, the maximum estimate for p-sites changed from 21,000 to 40,000 p-sites (potential HTP detection at 53%). The equivalent numbers for human are from 125,000 to 230,000 p-sites (potential HTP detection at 54%), whereas for mouse they are from 71,000 to 156,000 (potential HTP detection at 46%). This finding highlights the oft-neglected

importance of high-quality LTP studies and their expert annotation in specialized databases that may serve as 'gold standards' in the Omics era.

The most reliable estimates provided in our analysis are based on current datasets filtered and compiled from HTP phosphoproteomics and also on LTP, but highly confident experiments. Thus, it is conceivable that future HTP phosphoproteomic technologies/protocols may significantly change these estimates upwards, more probably for p-sites than for phosphoproteins. The more saturated the detection of phosphoproteins and p-sites, the less variability will be observed in the estimation of their total numbers with various methods and dataset manipulations/perturbations. However, our analysis provides a rigorous framework and a useful point of reference for all future updates on these estimates.

Potential Implications

Although HTP technologies will sooner or later mature to a level that allows the discovery of the total number of p-sites within a proteome, the real challenge that lies ahead is to determine which ones are noisy and which ones have a functional effect on phenotype [41–43]. Already, mutation studies of important p-sites in combination with proteomics and flux analysis or untargeted metabolomics show the way forward [44–46]. Considering the large number of p-sites estimated in this analysis, it is likely that such a daunting challenge will need to be addressed by a fusion of bioinformatics filtering analyses together with highly automated HTP omics and experimental processes that assess the phenotype of mutants [47,48].

Methods.

The Capture-Recapture method.

This method is widely used in epidemiology and ecology for estimating unknown population sizes and has been implemented in the R software package as the Rcapture module [49]. It has also been implemented for the inference of protein count in MudPIT experiments and for the inference of human p-sites based on data from two databases [9,50]. In this approach, the population under investigation is sampled several times and the observed pairwise overlap among the various samples is used to estimate the total population size. For our analysis, we assumed that the data resembled a closed population, meaning a finite and stable maximum number of phosphoproteins and p-sites. Another assumption was that the data were subject to temporal and contextual effects, meaning that the number of p-sites/phosphoproteins detected in the various experiments is not necessarily the same. A third assumption was that there is some heterogeneity among the different p-sites/phosphoproteins, implying that each p-site/phosphoprotein has its own probability of being captured/detected. This assumption is in accordance with a model of some proteins being expressed/phosphorylated most of the time, whereas other proteins are expressed/phosphorylated more transiently. Based on the Akaike information criterion test [51], embedded within the RCapture software, the Chao Mth model (M standing for model; t standing for temporal; h standing for heterogeneous) was selected for the subsequent analyses. The method is implemented in R and may input up to 15-20 different samples, depending on their size, as a matrix input file. Once the user loads the matrix input file in R, where each row represents a protein or p-site and each column represents an experimental dataset (with 0 for absence and 1 for presence), the Capture-recapture method is run by executing the “closedp(matrix)” function. Due to this limitation, original estimates for each species were based on the 15 largest datasets. In order to allow for variation, the Capture-Recapture analyses on human and mouse were repeated with a jackknife strategy, where only

datasets with 500 or more p-sites were retained. Next, within this retained subset, jackknife randomly selected 15 of those experiments and then calculated the population size, standard deviation, and coefficient of variation. This jackknife approach was repeated 100 times for each of the two species.

Estimation based on curve-fitting of data saturation.

The second method employed was based on graphing, in a given scatter plot, the cumulative number of non-redundant (unique) phosphoproteins/p-sites (y-axis of a given scatter plot) identified as relevant experiments accumulated over time against the cumulative number of redundant p-sites/proteins (x-axis of a given scatter plot). In essence, it constitutes a visualization of the saturation level of the experiments. For example, if one assumes that one experiment identifies 1,000 p-sites; then, up to this point, the total number of unique p-sites is 1,000. A second experiment identifies 900 p-sites, but 100 of those were identified previously. Therefore, at the time of the second experiment, 1,900 redundant p-sites have accumulated (x-axis of a given scatter plot), whereas the cumulative number of unique p-sites now rises to 1,800 (y-axis of a given scatter plot). In such a process, the cumulative number of non-redundant units (phosphoproteins or p-sites) rises steeply at the beginning and very slowly later as more and more experiments accumulate. The cumulative number of units will converge to a plateau value that approximates the total number of units in that proteome. This process is best modeled by an exponential recovery curve. Indeed, simulations of such a process verified the exponential nature of recovery, which is modeled by equation 1:

$$y=a*(1 - e^{(-x/b)})$$

In this equation, **x** is the cumulative number of redundant units (p-sites/phosphoproteins), y is the cumulative number of non-redundant units that have been identified up to that point, **a** is a constant that reveals the maximum value of y (that is actually the estimated total number of non-redundant units) and **b** is a constant that defines the steepness of the curve and is the total number of redundant units needed to be detected in order to identify 63.2% of total non-redundant units. Estimation of the above parameters was performed with Curve-Fitting in Microsoft Excel, by optimizing the **a** and **b** parameters with the GRG non-linear solving method, in order to minimize the sum of squared errors (SSE) between the observed and theoretical values. The curve-fitting process is explained in detail in supplementary file S6, which is a screencasting mp4 video.

Controlling for various confounding factors

Highly similar experimental datasets may artificially inflate the observed saturation of the sampled population. Therefore, it is necessary to assess the level of pairwise overlap among the various experiments to exclude any highly similar datasets. In order to achieve this, the Jaccard distance and similarity (1 – Jaccard distance) between all pairs of experiments (within a species) was estimated with the pdist function in Matlab. This distance is used for binary variables (in this case, 1 and 0 for detection or non-detection of phosphorylation of a protein/site in a certain experiment). This distance is the quotient between the intersection and the union between two experiments. The results of the Jaccard distance between the various experiments is summarized in supplementary excel file S7. For proteins, the average and maximum Jaccard similarities ranged between 0.07 - 0.24 and 0.51 – 0.63,

respectively. For p-sites, the average and maximum Jaccard similarity ranged between 0.04 – 0.09 and 0.27 – 0.5 respectively. Thus, all experiments were included in subsequent analyses.

Any HTP experiment is susceptible to noise and phosphoproteomics is no exception. Furthermore, current analyses of mass spectra are usually performed automatically, by algorithms with varying probabilities of error. The phosphoproteomic experiments that were used in this study were further filtered with a cut-off of 99% correct phosphopeptide identification and 99% correct p-site localization. However, these are values provided by the various phosphoproteomic software packages.

In order to model the effect of noise on our estimates, three basic assumptions were made: i) noise has a stochastic nature; ii) the pool of noise (potential false-positive p-sites and phosphoproteins) is large; iii) the level of noise within a given experiment is relatively low. Assuming that the above three assumptions are reasonably valid, it is expected that the overlap of false-positive p-sites/phosphoproteins among the various experiments is very small, if not negligible.

For the Capture-Recapture algorithm, the presence of such noise is expected to cause the algorithm to overestimate the total number of p-sites, due to the presence of non-overlapping false-positive identifications in the various datasets. In order to re-adjust the estimates, 1%, 5%, and 10% more noise was added to all the current datasets and the consequent increase in the estimates made by the algorithm determined. Based on the results from this artificial increase, an appropriate downward adjustment of the original estimates was made, for each particular level of noise.

The effect of noise can be modeled in the curve-fitting approach as well. Here, the number of false-positives in the compendium will increase for some time in a linear fashion (due to negligible overlap). Thus, while the number of experiments continues to increase, the number of new true-positives will plateau, whereas noise will cause false-positives to continue to accumulate in a linear fashion, as shown in equation 2:

$$\mathbf{y=a*(1-e^{(-x/b)}) + c*x},$$

where: **c** is now the average noise level within the experiments.

It is conceivable that the curve-fitting estimates may be affected by the order in which the experiments were performed (or, at least, published). To control for such a possibility, the order of the experiments was changed in two ways, such that the largest experiment was placed either first or last in the temporal order, and the parameters of the curve re-calculated. In addition, the curve-fitting parameters were recalculated, but only for the earlier half of the experiments on each species. In these ways, it is possible to determine the extent to which the estimates are affected by the temporal ordering of the experiments and thus assess their robustness.

Evaluation of the two methods on the yeast proteome.

In order to assess how reliable are the two methods of Capture-Recapture and Curve-Fitting of the saturation curve, estimates were calculated for a published proteomics experiment with a known outcome. Five proteomics experiments were performed for yeast [33], during normal growth conditions and by using five different proteases for peptide cleavage, ArgC, GluC, LysC, AspN,

Trypsin. Each of the five experiments identified between 2674 – 3264 yeast proteins. By combining all five datasets, these protocols were capable of identifying ~3900 yeast protein, out of an estimated (based on GFP and TAP-tag) 4500, expressed in normal growth conditions [52]. In our analysis, each of the five experiments was randomly downsampled to 50%. After downsampling, the RCapture method estimated a total of 3805 proteins, whereas the Curve-fitting of the saturation curve estimated a total of 3732 proteins, which were very close to the total identification of 3908 proteins, based on these HTP technologies. Results are shown in Supplementary file S8.

Availability of supporting data

All supplementary data can be downloaded from our laboratory website:

http://bioinf.bio.uth.gr/total-phosphoproteome-estimate.html

The phosphorylation sites for each of the four species are organized in separate files (S1-S4) in csv format, where each row corresponds to the protein and the phosphorylated aminoacid (numbered) and each column corresponds to the published dataset (Pubmed ID in the first row) that it was detected as phosphorylated. All articles that were used to extract data are found in Supplementary excel file S5. The Supplementary Mp4 video (S6) demonstrates the implementation of Curve-fitting in Microsoft Excel. Of note, the Solver add-in needs to be installed first. The supplementary excel file S7 contains the results of the Jaccard distance between the various datasets of each species. The supplementary excel file S8 contains the evaluation of the Capture-Recapture and Curve-fitting on the five yeast proteomics experiments (five proteases) (each randomly downsampled at 50%) of Swaney et al., 2010. The supporting data associated with this manuscript is also openly available in the *GigaScience* repository, GigaDB [53]

Abbreviations

p-site – phosphorylation site; phosphoprotein – phosphorylated protein; HTP – High ThroughPut; LTP – Low ThroughPut

Declarations

Funding

G.D.A. acknowledges financial support from the "ARISTEIA II" Action of the "OPERATIONAL PROGRAMME EDUCATION AND LIFELONG LEARNING" that is co-funded by the European Social Fund (ESF) and National Resources (code 4288 to G.D.A.). G.D.A. acknowledges additional support by research grants from the Postgraduate Programme 'Toxicology', of the Dept. of Biochemistry and Biotechnology, School of Health Sciences, University of Thessaly, Greece. Y.V.d.P. acknowledges the Multidisciplinary Research Partnership "Bioinformatics: from nucleotides to networks" Project (no. 01MR0310W) of Ghent University. S.G.O. acknowledges the University of Cambridge for granting him Sabbatical Leave to permit him to work with G.D.A. in the University of Thessaly, Greece.

Author's contributions

P.V., P.K, A.C, G.D.A gathered, filtered and analyzed data. Y.Vd.P, S.G.O and G.D.A conceived the study and wrote the paper. G.D.A supervised the project.

Competing interests: None declared

References

1. Krüger R, Kübler D, Pallissé R, Burkovski A, Lehmann WD. Protein and proteome phosphorylation stoichiometry analysis by element mass spectrometry. Anal. Chem. 2006;78:1987–94.

2. Nishi H, Shaytan A, Panchenko AR. Physicochemical mechanisms of protein regulation by phosphorylation. Front Genet. 2014;5:270.

3. Cohen P. The regulation of protein function by multisite phosphorylation--a 25 year update. Trends Biochem. Sci. 2000;25:596–601.

4. Amoutzias GD, He Y, Gordon J, Mossialos D, Oliver SG, Van de Peer Y. Posttranslational regulation impacts the fate of duplicated genes. Proc. Natl. Acad. Sci. U.S.A. 2010;107:2967–71.

5. Amoutzias GD, He Y, Lilley KS, Van de Peer Y, Oliver SG. Evaluation and properties of the budding yeast phosphoproteome. Mol. Cell Proteomics. 2012;11:M111.009555.

6. Cohen P. The origins of protein phosphorylation. Nat. Cell Biol. 2002;4:E127-130.

7. Pinna LA, Ruzzene M. How do protein kinases recognize their substrates? Biochim. Biophys. Acta. 1996;1314:191–225.

8. Sadowski I, Breitkreutz B-J, Stark C, Su T-C, Dahabieh M, Raithatha S, et al. The PhosphoGRID Saccharomyces cerevisiae protein phosphorylation site database: version 2.0 update. Database (Oxford). 2013;2013:bat026.

9. Boekhorst J, Boersema PJ, Tops BBJ, van Breukelen B, Heck AJR, Snel B. Evaluating experimental bias and completeness in comparative phosphoproteomics analysis. PLoS ONE. 2011;6:e23276.

10. Boersema PJ, Foong LY, Ding VMY, Lemeer S, van Breukelen B, Philp R, et al. In-depth qualitative and quantitative profiling of tyrosine phosphorylation using a combination of phosphopeptide immunoaffinity purification and stable isotope dimethyl labeling. Mol. Cell Proteomics. 2010;9:84–99.

11. Lemeer S, Heck AJR. The phosphoproteomics data explosion. Curr Opin Chem Biol. 2009;13:414–20.

12. Ubersax JA, Ferrell JE. Mechanisms of specificity in protein phosphorylation. Nat. Rev. Mol. Cell Biol. 2007;8:530–41.

13. Sharma K, D'Souza RCJ, Tyanova S, Schaab C, Wiśniewski JR, Cox J, et al. Ultradeep human phosphoproteome reveals a distinct regulatory nature of Tyr and Ser/Thr-based signaling. Cell Rep. 2014;8:1583–94.

14. Cunningham F, Amode MR, Barrell D, Beal K, Billis K, Brent S, et al. Ensembl 2015. Nucleic Acids Research. 2015;43:D662–9.

15. Lamesch P, Berardini TZ, Li D, Swarbreck D, Wilks C, Sasidharan R, et al. The Arabidopsis Information Resource (TAIR): improved gene annotation and new tools. Nucleic Acids Research. 2012;40:D1202–10.

16. Costanzo MC, Engel SR, Wong ED, Lloyd P, Karra K, Chan ET, et al. Saccharomyces genome database provides new regulation data. Nucleic Acids Research. 2014;42:D717–25.

17. Hornbeck PV, Zhang B, Murray B, Kornhauser JM, Latham V, Skrzypek E. PhosphoSitePlus, 2014: mutations, PTMs and recalibrations. Nucleic Acids Res. 2015;43:D512-520.

18. Stark C, Su T-C, Breitkreutz A, Lourenco P, Dahabieh M, Breitkreutz B-J, et al. PhosphoGRID: a database of experimentally verified in vivo protein phosphorylation sites from the budding yeast Saccharomyces cerevisiae. Database (Oxford). 2010;2010:bap026.

19. Goffeau A, Barrell BG, Bussey H, Davis RW, Dujon B, Feldmann H, et al. Life with 6000 genes. Science. 1996;274:546, 563–7.

20. Oliver SG, van der Aart QJ, Agostoni-Carbone ML, Aigle M, Alberghina L, Alexandraki D, et al. The complete DNA sequence of yeast chromosome III. Nature. 1992;357:38–46.

21. de Godoy LMF, Olsen JV, Cox J, Nielsen ML, Hubner NC, Fröhlich F, et al. Comprehensive mass-spectrometry-based proteome quantification of haploid versus diploid yeast. Nature. 2008;455:1251–4.

22. Wu R, Dephoure N, Haas W, Huttlin EL, Zhai B, Sowa ME, et al. Correct interpretation of comprehensive phosphorylation dynamics requires normalization by protein expression changes. Mol. Cell Proteomics. 2011;10:M111.009654.

23. Beltrao P, Trinidad JC, Fiedler D, Roguev A, Lim WA, Shokat KM, et al. Evolution of phosphoregulation: comparison of phosphorylation patterns across yeast species. PLoS Biol. 2009;7:e1000134.

24. Albuquerque CP, Smolka MB, Payne SH, Bafna V, Eng J, Zhou H. A multidimensional chromatography technology for in-depth phosphoproteome analysis. Mol. Cell Proteomics. 2008;7:1389–96.

25. Choudhary G, Wu S-L, Shieh P, Hancock WS. Multiple enzymatic digestion for enhanced sequence coverage of proteins in complex proteomic mixtures using capillary LC with ion trap MS/MS. J. Proteome Res. 2003;2:59–67.

26. Gauci S, Helbig AO, Slijper M, Krijgsveld J, Heck AJR, Mohammed S. Lys-N and trypsin cover complementary parts of the phosphoproteome in a refined SCX-based approach. Anal. Chem. 2009;81:4493–501.

27. Lee DCH, Jones AR, Hubbard SJ. Computational phosphoproteomics: from identification to localization. Proteomics. 2015;15:950–63.

28. Wiśniewski JR, Mann M. Consecutive proteolytic digestion in an enzyme reactor increases depth of proteomic and phosphoproteomic analysis. Anal. Chem. 2012;84:2631–7.

29. Hedges SB, Dudley J, Kumar S. TimeTree: a public knowledge-base of divergence times among organisms. Bioinformatics. 2006;22:2971–2.

30. Clamp M, Fry B, Kamal M, Xie X, Cuff J, Lin MF, et al. Distinguishing protein-coding and noncoding genes in the human genome. Proc. Natl. Acad. Sci. U.S.A. 2007;104:19428–33.

31. Church DM, Goodstadt L, Hillier LW, Zody MC, Goldstein S, She X, et al. Lineage-specific biology revealed by a finished genome assembly of the mouse. PLoS Biol. 2009;7:e1000112.

32. Vlastaridis P, Oliver SG, Van de Peer Y, Amoutzias GD. The Challenges of Interpreting Phosphoproteomics Data: A Critical View Through the Bioinformatics Lens. In: Angelini C, Rancoita PM, Rovetta S, editors. Computational Intelligence Methods for Bioinformatics and Biostatistics [Internet]. Cham: Springer International Publishing; 2016 [cited 2016 Nov 29]. p. 196–204. Available from: http://link.springer.com/10.1007/978-3-319-44332-4_15

33. Swaney DL, Wenger CD, Coon JJ. Value of using multiple proteases for large-scale mass spectrometry-based proteomics. J. Proteome Res. 2010;9:1323–9.

34. Tsiatsiani L, Giansanti P, Scheltema RA, van den Toorn H, Overall CM, Altelaar AFM, et al. Opposite Electron-Transfer Dissociation and Higher-Energy Collisional Dissociation Fragmentation Characteristics of Proteolytic K/R(X) $_n$ and (X) $_n$ K/R Peptides Provide Benefits for Peptide Sequencing in Proteomics and Phosphoproteomics. Journal of Proteome Research [Internet]. 2016 [cited 2016 Dec 6]; Available from: http://pubs.acs.org/doi/abs/10.1021/acs.jproteome.6b00825

35. Giansanti P, Tsiatsiani L, Low TY, Heck AJR. Six alternative proteases for mass spectrometry-based proteomics beyond trypsin. Nat Protoc. 2016;11:993–1006.

36. Iakoucheva LM, Radivojac P, Brown CJ, O'Connor TR, Sikes JG, Obradovic Z, et al. The importance of intrinsic disorder for protein phosphorylation. Nucleic Acids Res. 2004;32:1037–49.

37. Moses AM, Hériché J-K, Durbin R. Clustering of phosphorylation site recognition motifs can be exploited to predict the targets of cyclin-dependent kinase. Genome Biol. 2007;8:R23.

38. Schweiger R, Linial M. Cooperativity within proximal phosphorylation sites is revealed from large-scale proteomics data. Biol. Direct. 2010;5:6.

39. Bodenmiller B, Mueller LN, Mueller M, Domon B, Aebersold R. Reproducible isolation of distinct, overlapping segments of the phosphoproteome. Nat. Methods. 2007;4:231–7.

40. Fíla J, Honys D. Enrichment techniques employed in phosphoproteomics. Amino Acids. 2012;43:1025–47.

41. Lienhard GE. Non-functional phosphorylations? Trends Biochem. Sci. 2008;33:351–2.

42. Landry CR, Freschi L, Zarin T, Moses AM. Turnover of protein phosphorylation evolving under stabilizing selection. Front Genet. 2014;5:245.

43. Landry CR, Levy ED, Michnick SW. Weak functional constraints on phosphoproteomes. Trends Genet. 2009;25:193–7.

44. Oliveira AP, Ludwig C, Picotti P, Kogadeeva M, Aebersold R, Sauer U. Regulation of yeast central metabolism by enzyme phosphorylation. Mol. Syst. Biol. 2012;8:623.

45. Oliveira AP, Sauer U. The importance of post-translational modifications in regulating Saccharomyces cerevisiae metabolism. FEMS Yeast Res. 2012;12:104–17.

46. Raguz Nakic Z, Seisenbacher G, Posas F, Sauer U. Untargeted metabolomics unravels functionalities of phosphorylation sites in Saccharomyces cerevisiae. BMC Syst Biol. 2016;10:104.

47. King RD, Rowland J, Oliver SG, Young M, Aubrey W, Byrne E, et al. The automation of science. Science. 2009;324:85–9.

48. King RD, Whelan KE, Jones FM, Reiser PGK, Bryant CH, Muggleton SH, et al. Functional genomic hypothesis generation and experimentation by a robot scientist. Nature. 2004;427:247–52.

49. Baillargeon S, Rivest L-P. The Rcapture Package: Loglinear Models for Capture-Recapture in R. Journal of Statistical Software [Internet]. 2007 [cited 2016 Mar 13];19. Available from: http://www.jstatsoft.org/v19/i05/

50. Koziol JA, Feng AC, Schnitzer JE. Application of capture-recapture models to estimation of protein count in MudPIT experiments. Anal. Chem. 2006;78:3203–7.

51. Akaike H. A new look at the statistical model identification. IEEE Transactions on Automatic Control. 1974;19:716–23.

52. Ghaemmaghami S, Huh W-K, Bower K, Howson RW, Belle A, Dephoure N, et al. Global analysis of protein expression in yeast. Nature. 2003;425:737–41.

53. Vlastaridis P, Kyriakidou P, Chaliotis A, de Peer YV, Oliver SG, Amoutzias GD: Supporting data for "Estimating the total number of phosphoproteins and phosphorylation sites in eukaryotic proteomes." GigaScience Database. 2016. http://dx.doi.org/10.5524/100267

Figures legends.

Figure 1. Estimation of the total number of phosphoproteins (1A, 1B) and p-sites (1C, 1D) for yeast, with the curve-fitting (assuming 1% noise) and Capture-Recapture methods, also correcting for 3 levels of noise (1%, 5%, 10%). In figures 1A and 1C, the x-axis is the cumulative number of redundant phosphoproteins/p-sites, whereas the y-axis is the cumulative number of non-redundant phosphoproteins/p-sites. The red curve is fitted for 1% noise. In figures 1B and 1D: Current is the total number of phosphoproteins/p-sites detected so far (by applying our filtering criteria). Current_3X is the total number of phosphoproteins/p-sites detected so far in at least 3 experiments. Rcapture is the estimation of maximum number of phosphoproteins/p-sites based on the Rcapture method (using the 15 largest datasets). Rcapture_HTP_vs_LTP is the estimation of maximum number of phosphoproteins/p-sites based on the Rcapture method, but this time using only two datasets, where one of them is the compendium of all HTP experiments and the second is the compendium of all LTP experiments from PhosphoGrid2. CF is the estimation of maximum number of phosphoproteins/p-sites based on the curve-fitting method of the saturation curve from all experiments. CF_3X is the estimation of maximum number of phosphoproteins/p-sites identified in at least 3 experiments, based on the curve-fitting method (in this case, a reasonable estimate was not possible). CF_best_start is the estimation of maximum number of phosphoproteins/p-sites based on the curve-fitting method of the saturation curve from all experiments, but this time, the largest experiment is used as first in the series. CF_best_end is the estimation of maximum number of phosphoproteins/p-sites based on the curve-fitting method of the saturation curve from all experiments, but this time, the largest experiment is used as last in the series. CF_half_exp is the estimation of maximum number of phosphoproteins/p-sites based on the curve-fitting method of the saturation curve from the first half experiments.

Figure 2. Estimation of the number of phosphoproteins (2A, 2B) and p-sites (2C, 2D) for human, with the Curve-Fitting (assuming 1% noise) and Capture-Recapture methods, also correcting for various levels of noise (1%, 5%, 10%). See legend of Figure 1 for explanations.

Figure 3. Estimation of the number of phosphoproteins (3A, 3B) and p-sites (3C, 3D) for mouse, with the Curve-Fitting (assuming 1% noise) and Capture-Recapture methods, also correcting for 3 levels of noise (1%, 5%, 10%). See legend of figure 1 for explanations. Estimates on figures 3B and 3D are obtained for a Vega annotated proteome of 16,000 protein-coding genes, where all estimates have been readjusted 25% upwards.

Figure 4. Estimation of the number of phosphoproteins (4A, 4B) and p-sites (4C, 4D) for *Arabidopsis*, with the Curve-Fitting (assuming 1% noise) and Capture-Recapture methods, also correcting for 3 levels of noise (1%, 5%, 10%). See legend of Figure 1 for explanations.

Figure 1

Click here to download Figure Fig1_yeast.pdf

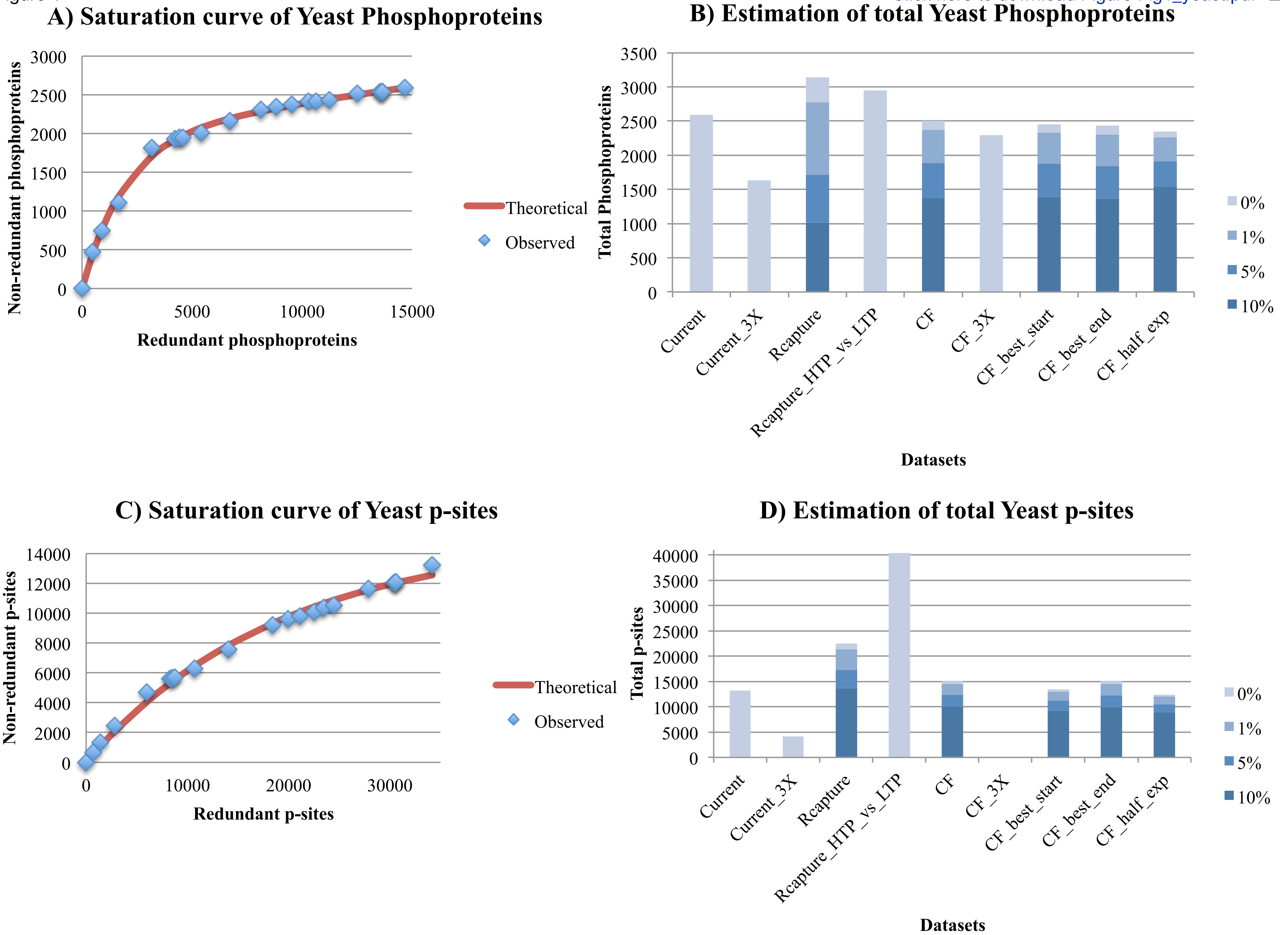

Figure 2

Click here to download Figure Fig2_human.pdf

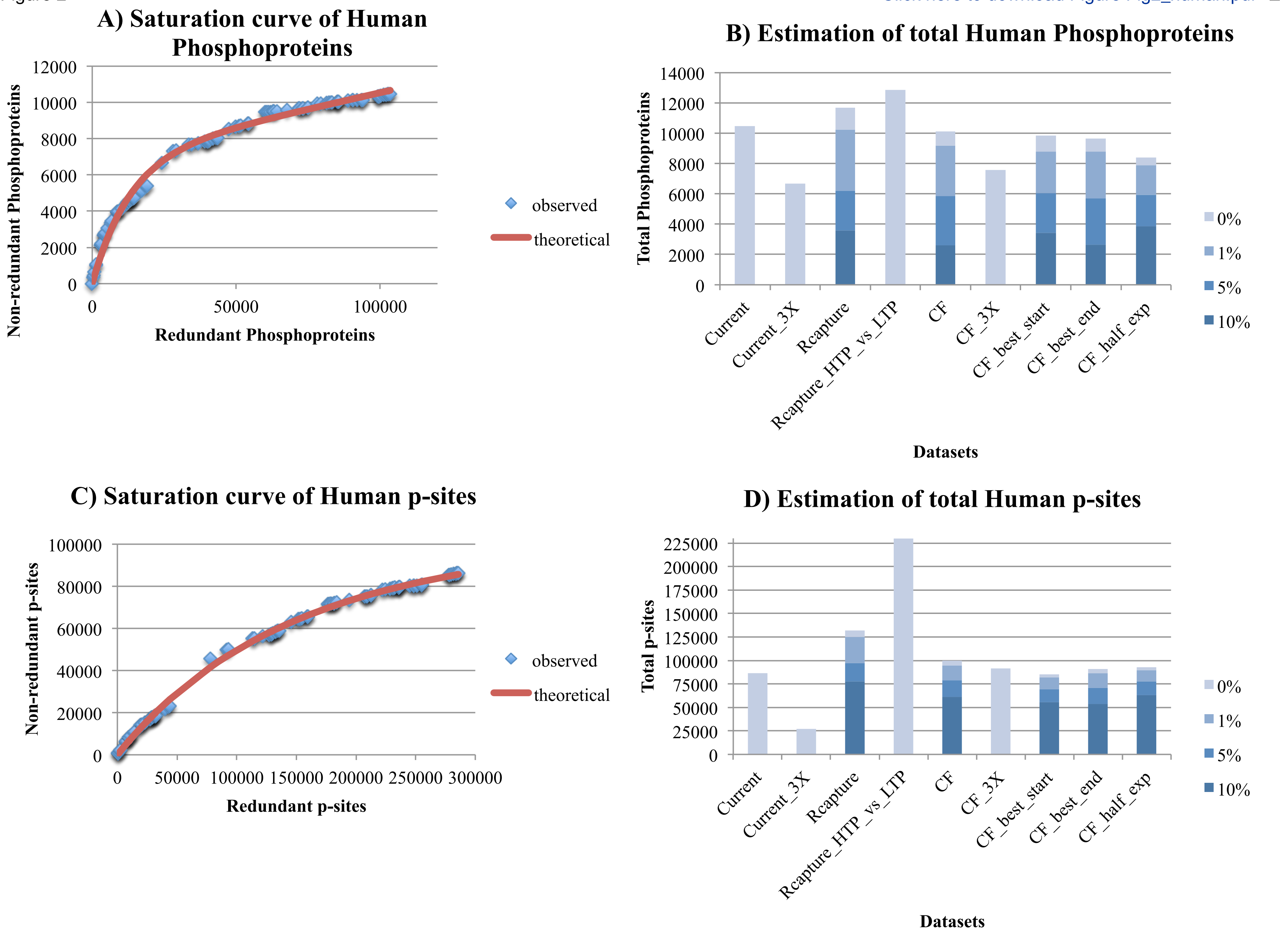

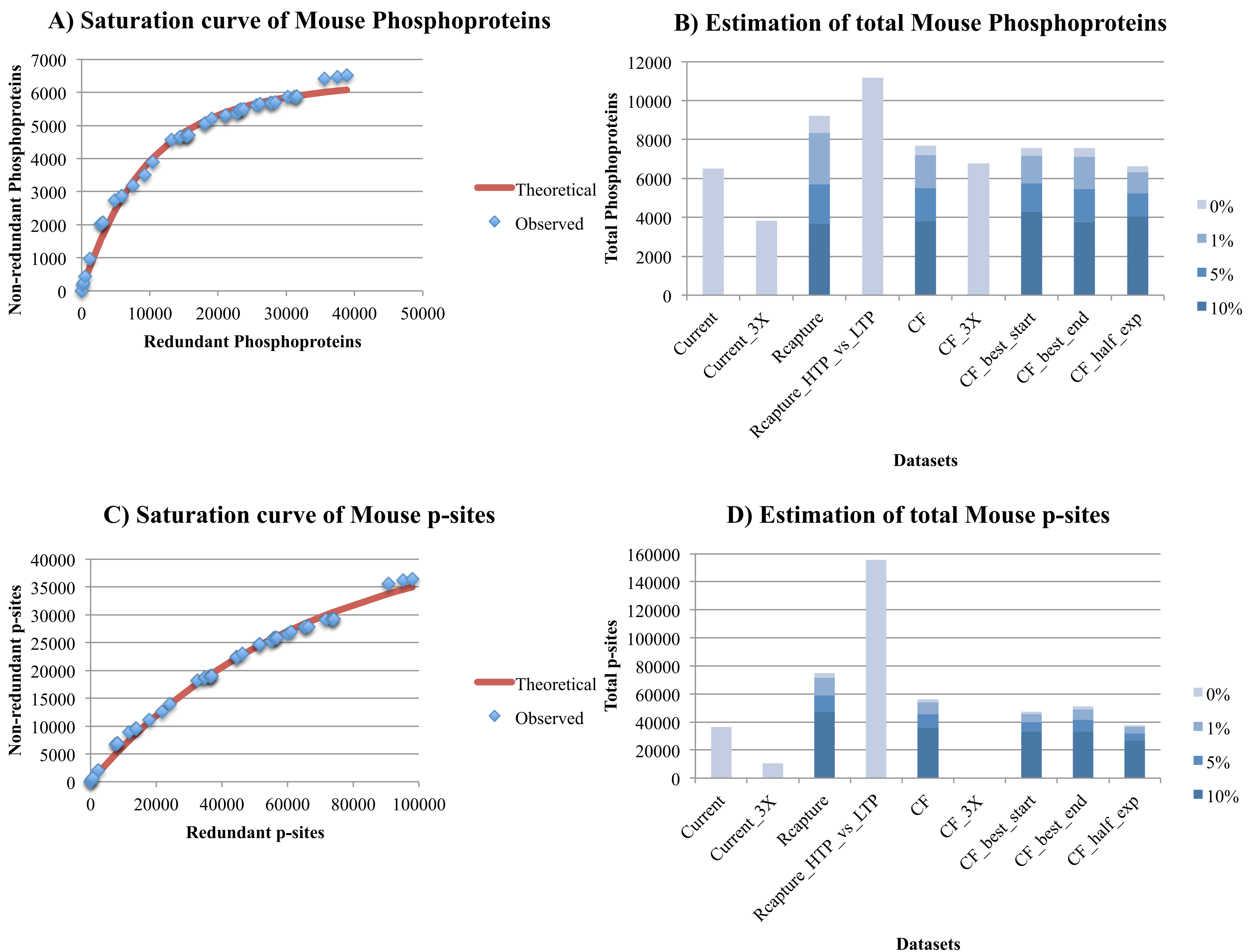
A) Saturation curve of Mouse Phosphoproteins
Non-redundant Phosphoproteins
Redundant Phosphoproteins
0
1000
2000
3000
4000
5000
6000
7000
0
10000
20000
30000
40000
50000
Theoretical
Observed
B) Estimation of total Mouse Phosphoproteins
Total Phosphoproteins
0
2000
4000
6000
8000
10000
12000
Current
Current_3X
Rcapture
Rcapture_HTP_vs_LTP
CF
CF_3X
CF_best_start
CF_best_end
CF_half_exp
Datasets
0%
1%
5%
10%
C) Saturation curve of Mouse p-sites
Non-redundant p-sites
Redundant p-sites
0
5000
10000
15000
20000
25000
30000
35000
40000
0
20000
40000
60000
80000
100000
Theoretical
Observed
D) Estimation of total Mouse p-sites
Total p-sites
0
20000
40000
60000
80000
100000
120000
140000
160000
Current
Current_3X
Rcapture
Rcapture_HTP_vs_LTP
CF
CF_3X
CF_best_start
CF_best_end
CF_half_exp
Datasets
0%
1%
5%
10%

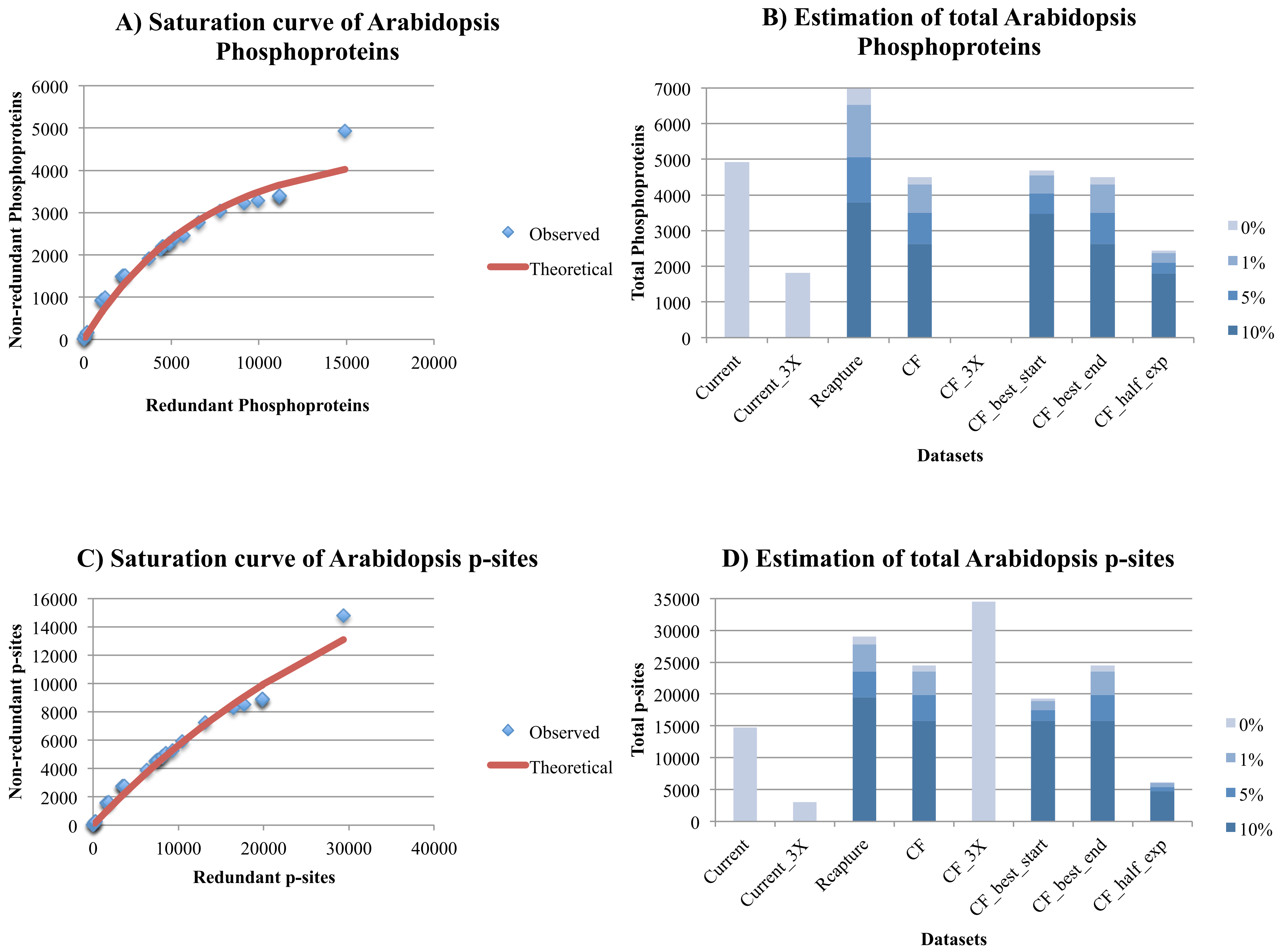
A) Saturation curve of Arabidopsis Phosphoproteins
Non-redundant Phosphoproteins
6000
5000
4000
3000
2000
1000
0
0
5000
10000
15000
20000
Redundant Phosphoproteins
Observed
Theoretical
B) Estimation of total Arabidopsis Phosphoproteins
Total Phosphoproteins
7000
6000
5000
4000
3000
2000
1000
0
Current
Current_3X
Rcapture
CF
CF_3X
CF_best_start
CF_best_end
CF_half_exp
Datasets
0%
1%
5%
10%
C) Saturation curve of Arabidopsis p-sites
Non-redundant p-sites
16000
14000
12000
10000
8000
6000
4000
2000
0
0
10000
20000
30000
40000
Redundant p-sites
Observed
Theoretical
D) Estimation of total Arabidopsis p-sites
Total p-sites
35000
30000
25000
20000
15000
10000
5000
0
Current
Current_3X
Rcapture
CF
CF_3X
CF_best_start
CF_best_end
CF_half_exp
Datasets
0%
1%
5%
10%

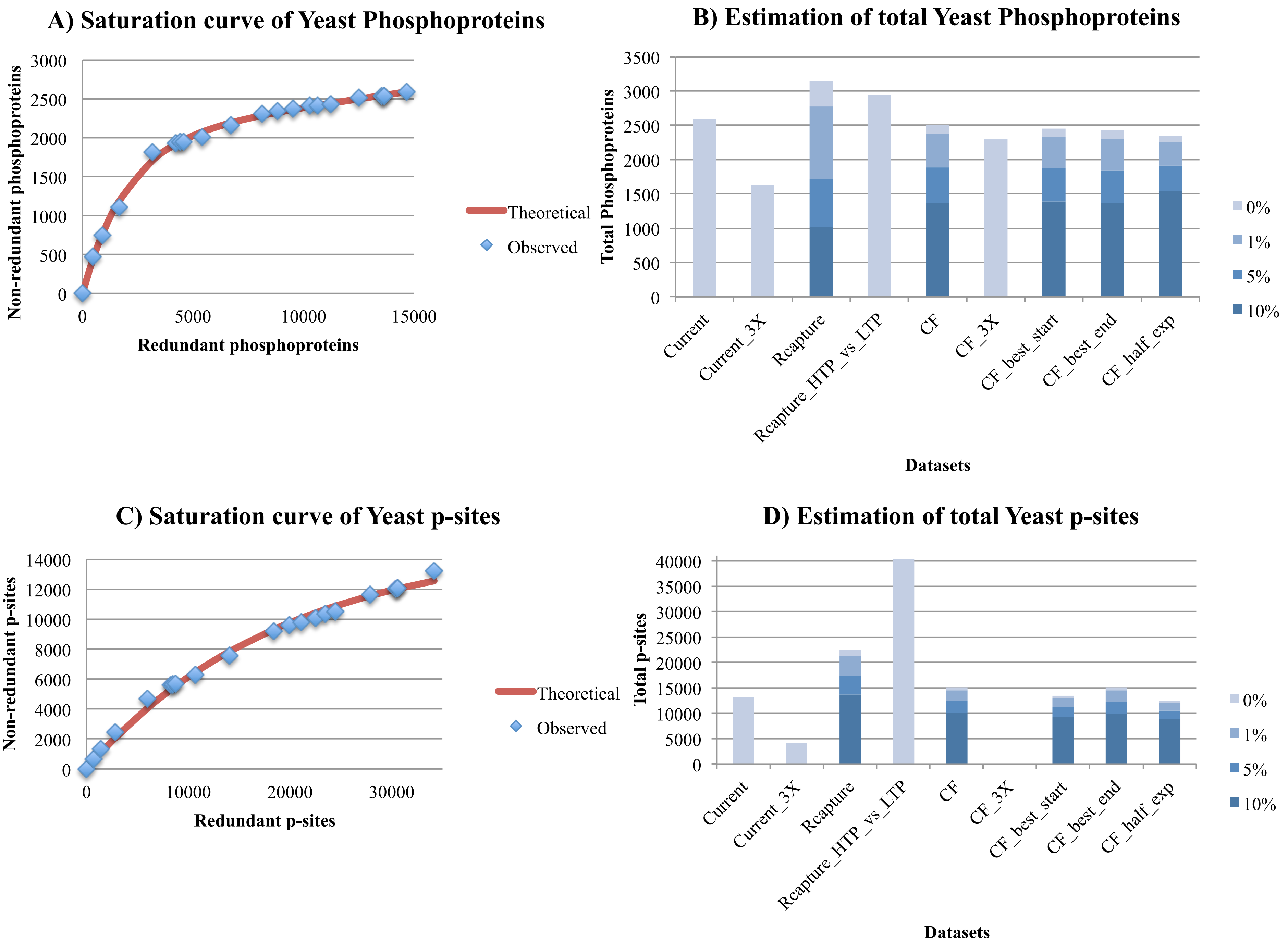
A) Saturation curve of Yeast Phosphoproteins
Non-redundant phosphoproteins
3000
2500
2000
1500
1000
500
0
0
5000
10000
15000
Redundant phosphoproteins
Theoretical
Observed
B) Estimation of total Yeast Phosphoproteins
Total Phosphoproteins
3500
3000
2500
2000
1500
1000
500
0
Current
Current_3X
Rcapture
Rcapture_HTP_vs_LTP
CF
CF_3X
CF_best_start
CF_best_end
CF_half_exp
Datasets
0%
1%
5%
10%
C) Saturation curve of Yeast p-sites
Non-redundant p-sites
14000
12000
10000
8000
6000
4000
2000
0
0
10000
20000
30000
Redundant p-sites
Theoretical
Observed
D) Estimation of total Yeast p-sites
Total p-sites
40000
35000
30000
25000
20000
15000
10000
5000
0
Current
Current_3X
Rcapture
Rcapture_HTP_vs_LTP
CF
CF_3X
CF_best_start
CF_best_end
CF_half_exp
Datasets
0%
1%
5%
10%

Figure 3

Click here to download Figure Fig3_mouse.pdf

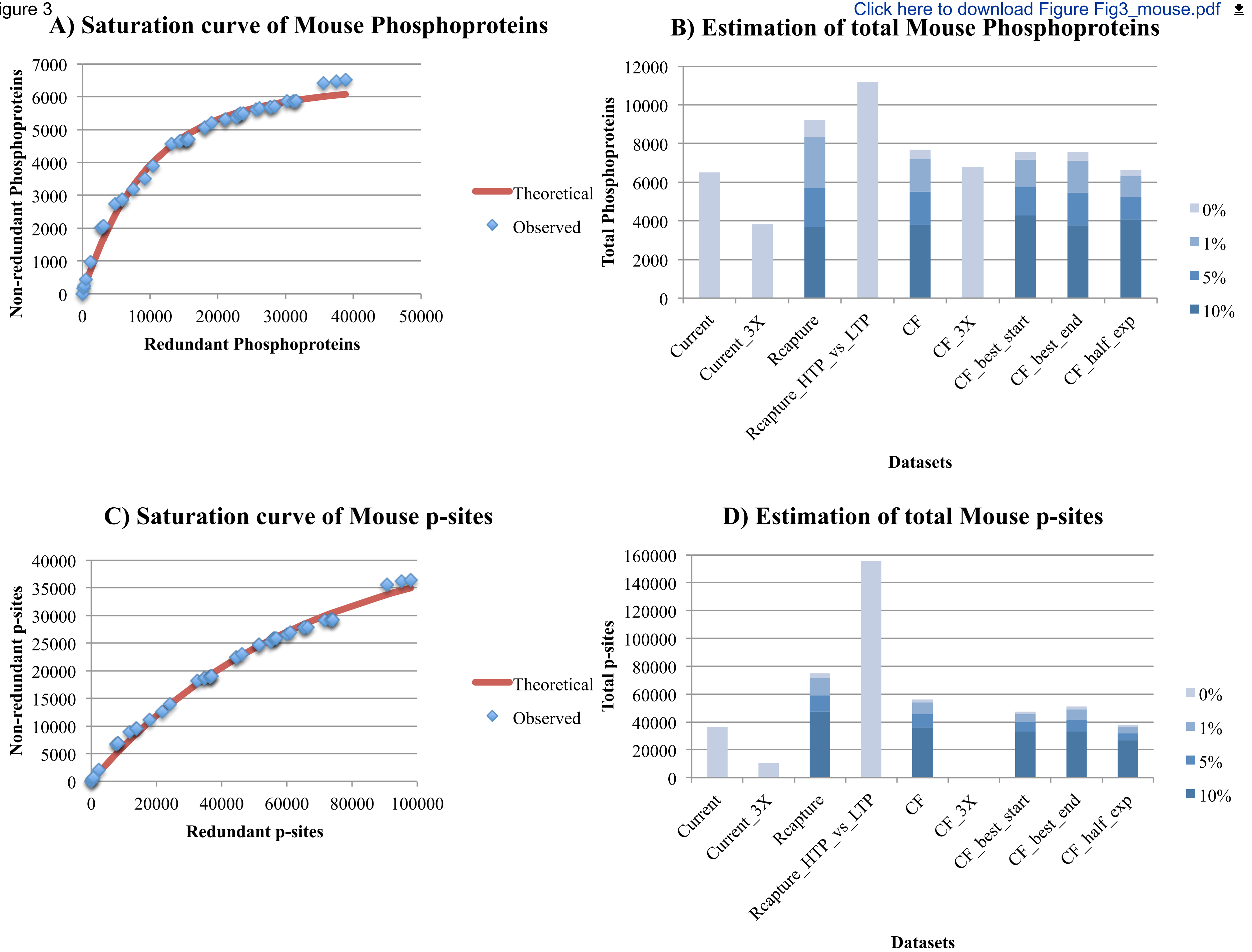

Figure 4

Click here to download Figure Fig4_arabidopsis.pdf

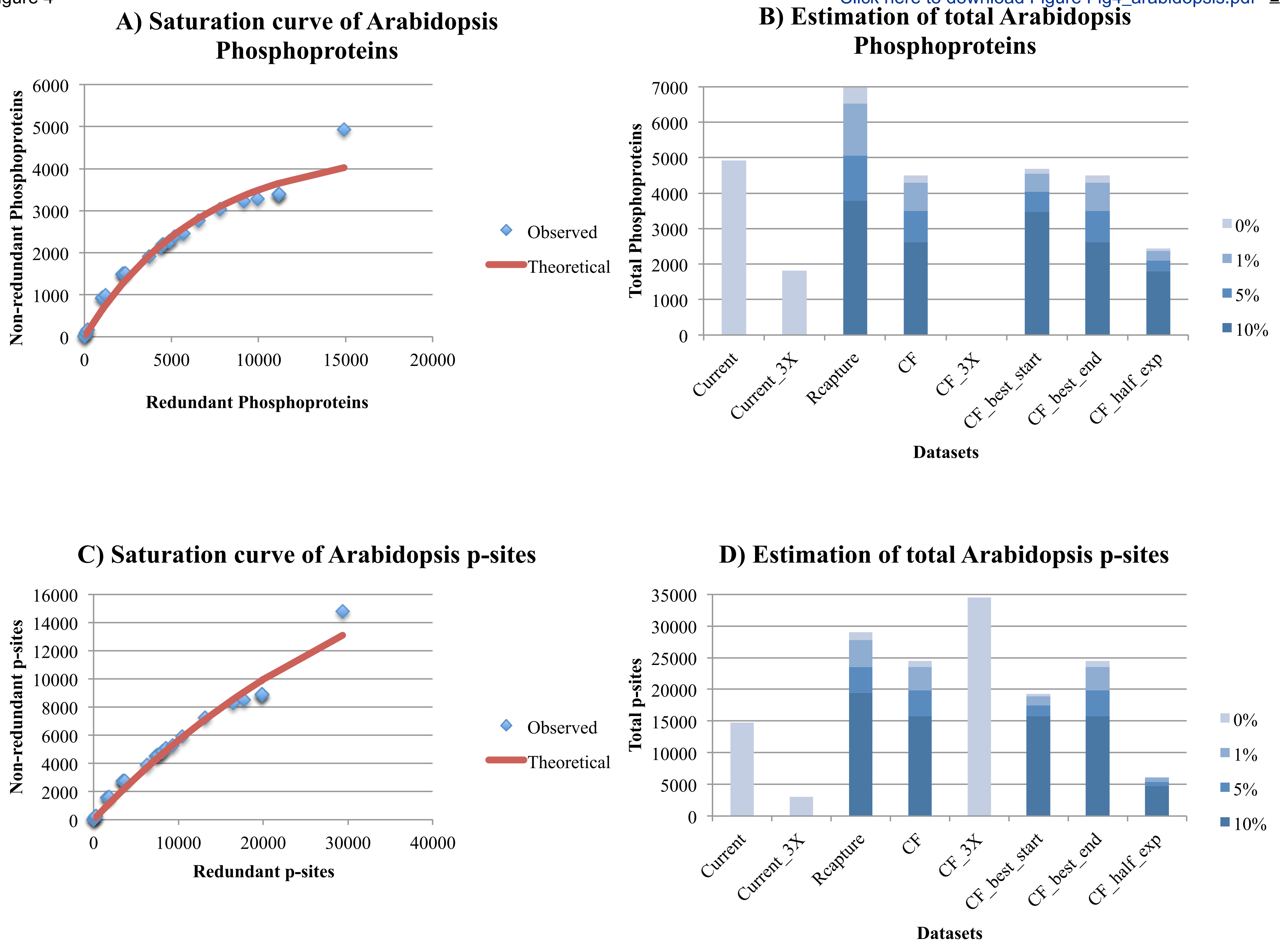

S1

Click here to access/download
Supplementary Material
S1_yeast_psites.csv

S2

Click here to access/download
Supplementary Material
S2_human_psites.csv

S3

Click here to access/download
Supplementary Material
S3_mouse_psites.csv

S4

Click here to access/download
Supplementary Material
S4_Arabidopsis_psites.csv

S5

Click here to access/download

Supplementary Material

S5_published_datasets.xlsx

S6

Click here to access/download
Supplementary Material
S6_GRG_minimization_curve_fitting.mp4

S7

Click here to access/download
Supplementary Material
S7_jaccard_distance_of_datasets.xlsx

S8

Click here to access/download
Supplementary Material
S8_Rcapture_CF_evaluation.xlsx
