# Supplementary material for: Estimating the total number of phosphoproteins and phosphorylation sites in eukaryotic proteomes
Source: Gigascience. 2017 Jan 7;6(2):1–11. doi: 10.1093/gigascience/giw015 (PMC5466708; doi:10.1093/gigascience/giw015)
Supplement: Response_to_Reviewer_Comments_Original_Submission.pdf [file giw015_response_to_reviewer_comments_original_submission.pdf]

Dear Dr. Nogoy

On behalf of all the authors, we would like to thank you for handling our manuscript (GIGA-D-16-00079) and for considering our work for publication in your journal. We would also like to thank the three referees, for their positive and constructive feedback on how to improve the manuscript. We appreciate their suggestions and thoughtful comments and we took full account of all the points raised that helped us to significantly improve our manuscript.

In an effort to improve the quality of our manuscript, we incorporated all the changes suggested by the referees and we addressed all the points raised, with all changes marked in yellow within the manuscript. In addition, we provide a detailed “Response to Referees” letter (see below), which accompanies our revised manuscript. We hope that our revised manuscript meets your requirements for final acceptance. We would very much appreciate it, if you could consider it for publication in Gigascience.

We look forward to hearing from you at your earliest convenience.

Sincerely,

Grigoris D. Amoutzias, Assistant Professor of Bioinformatics in Genomics, Department of Biochemistry and Biotechnology, University of Thessaly, Greece

Corresponding author

Reviewer #1

This is a well-written manuscript that reports on work estimating the number of phosphoproteins and phosphorylation sites from four eukaryotic organisms: yeast, mouse, human and Arabidopisis. A lot of manual work appears to have gone into this study to mine information about phosphoproteins and phosphorylation sites from over a 1000 articles which the authors responsible for doing this should be commended on. Since I only have a rudimentary knowledge of the biology of phosphoproteins, my comments relate to the reproducibility of the authors' work which is a focus of GigaScience.

Background

Referee: Line 73: From the 1000 plus articles that were surveyed, a set of 188 phosphoprotemic datasets were obtained and used for further analysis. The 188 datasets come from the papers listed in 5_published_datasets.txt - can the authors check that this file is referred to in the manuscript like the other supplementary files please?
Answer: This supplementary file is now an excel file designated as S5 and is described in supplementary data and in lines 471-472. We have also modified the sentence in lines 88-89 accordingly:
“Thus, 97, 42, 28 and 20 HTP datasets were retained for human, mouse, Arabidopsis, and budding yeast respectively (see supplementary files S1-S5).”

Referee: The 5_published_datasets.txt file lists 185 papers which I found a bit confusing as I wrongly assumed that there would be 188 papers relating to the 188 phosphoprotemic datasets. What appears to have happened is that 3 datasets were obtained from 1 of the yeast publications. A note should be added somewhere to report this detail.
Answer: We have now modified the following sentence in line 108 accordingly:
“Twenty HTP phosphoproteomic datasets from 18 articles have been collected from this organism,”

Referee: My text editor shows a strange character where a hyphen should be present to separate the starting and ending page numbers in the 5_published_datasets.txt file. Can the authors correct this problem if they see it too please?
Answer: We have now manually corrected this problem in supplementary file S5.

Referee: It would be good if the 5_published_datasets.txt file contains the Pubmed ID associated with each paper so that it can be cross-referenced with the *_psites.csv files.
Answer: The S5 file has now been renamed S5_published_datasets.xlsx and has incorporated the Pubmed ID of each article.

Data Description

Referee: Line 87 in the Data Description section says that 97, 42, 28 and 20 datasets were retained for human, mouse, Arabidopsis, and budding yeast, respectively. 97, 42, 28 and 20 equals 187 datasets but line 73 says 188 datasets were gathered. Can the authors confirm/correct the number of gathered datasets in the manuscript?
Answer: Indeed, 187 HTP datasets were gathered and this is corrected throughout the text (lines: 27, 74, 265)

Methods

Referee: Two methods were used to determine number of phosphorylated proteins. The capture-recapture method was done using R with the Rcapture module. It would be good if the authors provided access to the R script to allow readers to see how the capture-recapture method analysis was performed to promote reproducibility for readers.
Answer: We now explain in detail how the Capture-recapture method runs in R, by incorporating the following text in the RCapture method subsection of Methods section (lines: 359-363). Basically, its only one simple command: closedp(matrix).
"…as a matrix input file. Once the user loads the matrix input file in R, where each row represents a protein or p-site and each column represents an experimental dataset (with 0 for absence and 1 for presence), the Capture-recapture method is run by executing the "closedp(matrix)" function."

Referee: The curve-fitting on the saturation curve of redundant phosphoproteins/p-sites vs non-redundant phosphoproteins/p-sites is done in MS Excel using the GRG non-linear solving method. I would appreciate if the authors could look into performing this analysis in R to promote reproducibility. However, I am not sure that there is an R module which provides the GRG non-linear solving method for curve fitting - maybe there is information here: http://ebanshi.cc/questions/3525511/generalized-reduced-gradient-grg2-algorithm-in-r
Answer: We have now created a supplementary file, which is an mp4 screencasting video, named "S6_GRG_minimization_curve_fitting" that explains in detail how parameter optimization and curve-fitting were performed in Microsoft Excel. Also, we have added the following sentence in lines 398-399:
"The curve-fitting process is explained in detail in supplementary file S6, which is a screencasting mp4 video."

Referee: There is a MATLAB calculation (Jaccard distance and similarity) that measures the level of pairwise overlap between pairs of experiments so that highly similar datasets can be excluded in case they inflate the observed saturation of the sampled population. For the sake of completeness, it would be good to include this file and its data as supplementary materials.
Answer: We have now generated a supplementary excel file S7 that shows the Jaccard distance results for each species separately and have added the following sentence in lines 408-409:
"The results of the Jaccard distance between the various experiments is summarized in supplementary excel file S7."

Referee: I would like to suggest that all of the supplementary data files and scripts be made available in a GitHub repository with readme or documentation files. A link to the GitHub repository can then be referred to in the manuscript.
Answer: We have generated a webpage in our lab website that contains all the supplementary data, that we refer to in the document.
http://bioinf.bio.uth.gr/total-phosphoproteome-estimate.html
We have also modified the availability of supporting data section to include the download page URL and the description of the files (lines 465 - 477).

Discretionary revisions

Referee: Check if HTP and LTP need to be listed in abbreviations or written in full when first defined.
Answer: HTP and LTP are now included in abbreviations (lines 480-481), introduced for the first time in the abstract (line 22) and from that point on, they are always referred to by their abbreviation.

Referee: I would prefer to see labels for axes being present on the graphs themselves rather described in figure legends.
Answer: The new figures incorporate labels for their axes.

Reviewer #2

The authors estimate the number of phosphoproteins and phosphorylation sites in 4 model organisms (Human, Mouse, Arabidopsis and Yeast) and discuss the status of our current knowledge about phosphoproteins and phosphorylation sites.

In my opinion this work represents a valuable contribution to the field of phosphoproteomics. Indeed, it addresses two of the most frequently asked questions in the field: how many phosphoproteins and p-sites are present in model organisms? How many of them do we miss with the current technologies?

I only found two minor issues the authors must fix before submitting the final version of the manuscript:
Referee: #1 -- all figures: the axes are almost always not labeled. Although there is a description of each analysis above each graph and the variables are listed in the figure legends, I still feel that it is always a good practice to label the graphs.
Answer: The new figures incorporate labels for their axes.

Referee: #2 -- Figure 2: there are two labels ("CF_best_first", "CF_gest_last") that are never used elsewhere in the manuscript.
Answer: We meant CF_best_start and CF_best_end, which is now corrected in the new Figure 2.

I also have one small suggestion to improve the text:
Note for the authors: the line numbers I am referring to are those present in the original Word document.

Referee: #1 -- page 4, lines 76-77-- "we have obtained, for the first time, a reliable estimate of their total number for humans and three model eukaryotes.". I would say "we have obtained, for the first time, a reliable estimate of their total number for humans and three other model eukaryotes."
Answer: Done (lines 77-78)

Finally, I found a few typos the authors should correct before submitting the final version of the manuscript:
Referee: #1 -- page 1, line 22 -- "high throughput" => "high-throughput".
Answer: Done (line 22)

Referee: #2 -- page 6, line 127 -- "Fig, 1B" => "Figure 1B"
Answer: Done (line 131)

Referee: #3 -- page 6, line 131 -- "Figure1C" => "Figure 1C"
Answer:Done (line 134)

Referee: #4 -- page 7, lines 155-157 -- "In the Table, the most reliable estimates, obtained by incorporating both the HTP and LTP non-redundant datasets is highlighted in bold.". => "In the Table, the most reliable estimates, obtained by incorporating both the HTP and LTP non-redundant datasets are highlighted in bold.".
Answer: Done (line 159)

Referee: #5 -- page 10, line 191 -- "Jacknife" => "jackknife"
Answer: Done (line 193)

Referee: #6 -- page 11, lines 227-228 -- "lower than those of the human phosphoproteins and p-sites respectively. (See Table 1 for details.)" => "lower than those of the human phosphoproteins and p-sites respectively (See Table 1 for details)."
Answer: Done (lines 229-230)

Referee: #7 -- page 12, line 234 -- there are two typos. "14796" => "14,796"; "4930" => "4,930" -- check also the methods section.
Answer: These numbers (line 236) and all other numbers throughout the manunscript have now been checked/corrected for commas.

Referee:#8 -- page 12, lines 236-237 -- "while the estimates on their total number, based on the different methods and data treatments, is depicted in Figure 4B." => "while the estimates of their total number, based on the different methods and data treatments, are depicted in Figure 4B."
Answer:Done (line 239)

Referee:#9 -- page 26, line 549 -- "Racptture_HTP_vs_LTP" => "Rcapture_HTP_vs_LTP"
Answer:Done (line 642)

Referee:#10 -- page 26, line 554 -- "CF_3x" => "CF_3X"
Answer:Done (line 647)

Reviewer #3

In this study, the authors proposed a strategy to quantify the relative proportion of the phosphoproteins and phosphopeptide sites present in a few organisms. This is a very interesting question to tackle. The authors proposed two strategies which are: (1) Capture-Recapture and (2) fitting the saturation curve of cumulative redundant vs. cumulative non-redundant phosphoproteins/p-sites. This strategy, if valid, could be applied to other potential protein modifications as well. In addition to quantifying the completeness of the phosphoproteome being identified, the authors provide an exhaustive list of highly confident sites, which can serve as a valuable resource for any future studies and analyses. Therefore, I would like to see this manuscript and the list of highly confident phospho sites published and made available to the scientific community in some way. Yes this study is addressing an interesting question and the collaborative team proposing to
address the question seems highly capable; I highlight 2 main/major concerns and few minor points:

Referee: 1st concern: Need for validating the method. The authors proposed a strategy to answer a question which has an unknown answer, however it is common practice to validate any new strategy on a known dataset. Before testing the method to answer a question with no known answer, the method should be validated on a known proteomics system. Regarding a validation step, why not simply predict the size of a

proteome? The genome/proteome size of any model organism is roughly known and this can be used as a validation strategy. Using a small well characterised organism and possibly one organism that is not fully covered by proteomics experiments as well, it could be shown whether the two methods capture-recapture and fitting the saturation curve of cumulative redundant vs. cumulative non-redundant proteins are either close to or far from the predicted number of proteins.
Answer: The Capture-Recapture method is a well accepted method for estimating unknown population sizes, as we explain in the Methods section. We have now modified that sentence accordingly (line 344):
“This method is widely used in epidemiology and ecology for estimating unknown population sizes…”
We have also identified cases where this method was used in the proteomics community and have now added the following sentence with references in lines 345-347:
“It has also been implemented for the inference of protein count in MudPIT experiments and for the inference of human p-sites based on data from two databases…”
More importantly, we have performed an evaluation of the two methods on the datasets of a publication by Swaney et al., 2010. This analysis constitutes a new section in Methods, named: “Evaluation of the two methods on the yeast proteome” (lines 452-463). Five proteomics experiments were performed in yeast, during normal growth conditions and by using five different proteases for peptide cleavage, ArgC, GluC, LysC, AspN, Trypsin. By combining all five datasets, these protocols were capable of identifying ~3900 yeast protein. In our analysis, each of the five experiments was randomly downsampled to 50%. After downsampling, the RCapture method estimated a total of 3805 proteins, whereas the Curve-fitting of the saturation curve estimated a total of 3732 proteins, which were very close to the total identification of 3908 proteins. The results are shown in detail in the new supplementary excel file, S8.

Referee: 2nd concern: Addressing the bias peptide detection. Most of the phospho-proteomics studies (high or low throughput) have been performed using mainly 3 different enrichment methods, IMAC, titanium dioxide and p-Tyr pull down. While other variations do exist, those are the three most widely used methods. There is a small overlap between the outcome of those three methods which suggests that each of those methods have bias toward certain classes of phosphopeptides (this bias has been frequently reported, a recent review paper such as Fila & Honys, doi: 10.1007/s00726-011-1111-z mention it and this bias is an accepted fact). This could suggest the existence of a pool of phosphopeptides which can't be captured/detected using any of those three phospho-enrichment methods and will remain a fraction of false negatives. Most likely all detection methods will converge towards a specific value which is the "Detectable phosphoproteome".
Answer: We have now significantly revised the second paragraph of the Discussion section (lines 279-302), to clearly state the various concerns about experimental biases and false negatives. Concerning the above specific concern, we have added the following sentence in lines 292-295 of the second paragraph of the Discussion:
“Also, the vast majority of the phosphoproteomic datasets are generated by three enrichment methods (IMAC, TiO_2 and p-Tyr pull down) that are well known to exhibit relatively low overlap among them, due to inherent biases towards certain classes of phosphopeptides [39,40]”

Referee: What is the gap between the Complete phosphoproteome and the Detectable phosphoproteome? This is unknown and I am not sure how that pool of peptides could be tackled; perhaps using a complementary strategy combining absolute protein abundance evaluation with some 32P experiments? Here, I understand that addressing this question with a set of complex experiments is out of the scope of this study, however, the authors should be cautious in their statements being made.
Answer: Indeed, we provide approximate and not absolutely accurate estimates. That is the reason that we provided estimates from different dataset handlings in the four figures, to highlight the fluctuations and level of confidence. We have also modified the sentence in lines 274-277 accordingly:
“This analysis has generated what we believe is the first set of approximate estimates of the total number of phosphoproteins/p-sites in a range of species that is based on established computational and statistical approaches and which also critically assesses their validity.“

Referee: For example, in the abstract: "that current high-throughput phosphoproteomics is capable of capturing 70-95% of total phosphoproteins, but only 40-60% of total p-sites."

Answer: We have modified that sentence accordingly to highlight the caution on our estimates (line 38): "Integration of the LTP data suggests that current HTP phosphoproteomics appears to be capable of capturing 70-95% of total phosphoproteins, but only 40-60% of total p-sites."

Referee: I think the number of phospho-proteins shouldn't be greatly affected, however, the number of peptide p-sites are most likely underestimated. In addition to this enrichment method bias, there is also an underestimation of the number of phosphopeptides due to peptide detectability as any phosphopeptide level determination relies on a good protein coverage by LC-MS. A specific example
can be the protein Nanog in which there are some sites which are difficult to detect using standard phospho-proteomics due to their tryptic nature; for example one tryptic peptide stretch is a tryptic peptide of about 12kDa, potentially difficult to detect by LC-MS. In addition, I will digress a bit here; I haven't seen in the supplementary information any protein related to Nanog in mouse for example. Here, either I could have missed it or all of its reported sites didn't fit the high confidence threshold the authors used. Back to this pool of undetectable p-sites, I can imagine those false negatives will also contribute to underestimate the number of possible phosphosites but contrary to the method bias, it should be relatively "easy" for the authors to address this problem. A good strategy to estimate this number would be to determine the global proteome coverage, not in terms of the proteins identified but rather in terms of peptides covered by LC-MS. If we take yeast for example ; what is the overall coverage of the yeast peptidome itself? Assuming it is 80% (using a similar ID confidence used for the p-site), we can assume that the estimated phosphoproteome in this study should be 80% of the real value as the 20% refers to those peptides which are too short, too long, too hydrophobic, etc. to be detected. Assuming as well, a uniform distribution of the p-site across the proteome.
Answer: A strong point of our analysis was that the HTP p-sites were pooled as one experiment and the LTP p-sites from a gold-standard database such as Phosphosite Plus or PhosphoGrid2 were used as a second pool. Afterwards,, the HTP pool was compared against the LTP pool and with Capture-Recapture, the total p-sites were estimated. Therefore, this specific analysis overcomes to a large extent the biases and shortcomings of HTP technologies, because it used p-sites detected by LTP methods.
Interestingly, Swaney et al, 2010, showed that the use of additional proteases, apart from the standard Trypsin resulted in a higher proteomics coverage of ~35% of total Serines, Threonines, Tyrosines. We have now modified the Discussion section accordingly to incorporate this fact (lines 284 -286):
"Along the same line of evidence, a previous Proteomics analysis on yeast showed that the use of additional proteases, apart from the standard Trypsin resulted in a significant increase of proteomics coverage from 21% to 35% of total Serines, Threonines, Tyrosines [33]."
Yet, another fact that makes this question very difficult to answer is that the various peptides undergo an enrichment step, therefore, it is not (in our opinion) absolutely necessary that peptide coverage would reflect the phosphopeptide coverage. In, addition, things get even more complicated by the fact that peptide coverage is not uniform across the various datasets of an organism, but it evolves with time. Furthermore, it has been reported previously by several groups, including ours, that p-sites tend to form clusters, they are not evenly distributed across the proteome. We have added the following sentence in lines 289-292:
"P-sites are not evenly distributed across the proteome but tend to cluster, especially at disordered regions [5,36–38], thus increasing the probability of missing many neighboring p-sites due to problematic enzymatic digestion of that peptide region."

Referee:In conclusion, 2 different experimental factors contribute to reduce p-site detection:
1) Method Bias
2) The proportion of peptides that can be detected by mass spectrometry.
Suggested approach to tackle this problem: 1) Tone down any mention to "exact" quantitative phosphoproteome coverage by acknowledging the existence of a pool of non-detectable phosphopeptides (I would have coined that the "Dark-proteome" but the term has been already used for another reason). 2) Evaluate a global proteome coverage (using a similar stringency criterion to the one you use for your p-sites), to estimate the fraction of detectable proteome.
Answer:Please see above for extensive answers to both these issues. The first issue the referee highlights is now addressed by a much better revised second paragraph of the Dicussion section.
Regarding the second issue that the referee summarizes, we hope that we have discussed it in the best

possible manner.

Referee: 3rd concern (less major than the 2 previous one): Regarding the nature of the supplementary information. The Supplementary information tables are not too intuitive in their current format to be of use, unless you are an expert in database/bioinformatics.
In order to highlight the difficulty of using the data in that format, it took me some time to look at 3 sites and discover some key errors. There is an apparent systematic shift in the numbering of the amino acid phosphorylation sites. I only looked at 3 examples in yeast and none of them are correct:
YDR033W_288 is wrong and should be YDR033W_289
YDL051W_18 is wrong and should be YDL051W_19
YJL026W_14 is wrong and should be YJL026W_15
haven't checked the other species, but I strongly encourage the authors to correct this possible systematic error. If widespread, I would also encourage a "sanity check" such as randomly picking 500-1000 sites from their supplementary information and checking their correctness. This should also be added in the Methods section. The authors should double-check the other species and address this shift if it is has happened across all datasets being reported. I would consider the supplementary data publishable only after fixing this problem and validating them. I would have preferred that the following information be provided as well: the list of peptides themselves (tryptic or a 23 aa window centred on the p-site), their associated protein IDs and any form of confidence score (peptide identification and phosphosite assignment). While this last request is not absolutely necessary, it will contribute greatly to the data accessibility and usefulness to the wider scientific
community.
Answer: We would like to thank the reviewer for pinpointing the shift and for the 23 aa window suggestion. In supplementary files S1-S4 we now include the 23 aa window centered on the p-sites, which will actually make it easier for other readers to identify p-sites on the same proteins, but from different databases and accession numbers. We have also checked all species and corrected the shift. Also, we show the actual aminoacid, which is a sanity check of the data.

Minor points:
Referee: In data description: please provide the version/date of the different databases used.
Answer: Database versions are now added, in lines 90 and 92.

Referee: Figure 1,2,3 and 4, A and C please define the nature of your axis (i.e. an axis title).
Answer: Labels in axes have now been added.

Referee: Please ensure that all your acronyms are properly introduced at their first mention (HTP) line 105, LTP line 98
Answer: We have now introduced the two acronyms in the abstract (lines 22, 28) and also include them in abbreviations in lines 480-481.

Referee: Line 347 :''phosphoproteins/p-sites (y-axis) identified..'' Which y-axis figure are you referring too?. Idem at line 348, 353, etc… please refer to a SPECIFIC figure as it is not easy to guess here. If found elsewhere in the text, please ensure you present clearly the figure you are referring to.
Answer: Here, we mean in general, the x and y-axis of any given scatter plot, not a specific scatter plot in any of the manuscript figures. Accordingly, in that paragraph (lines 374, 376, 380, 381), every time we mention the x and y-axis, we now say “x-axis of a given scatter plot” and “y-axis of a given scatter plot”.
