# Supplementary material for: Estimating the total number of phosphoproteins and phosphorylation sites in eukaryotic proteomes
Source: Gigascience. 2017 Jan 7;6(2):1–11. doi: 10.1093/gigascience/giw015 (PMC5466708; doi:10.1093/gigascience/giw015)
Supplement: Reviewer_1_Report_(Original_Submission).pdf [file giw015_reviewer_1_report_(original_submission).pdf]

Reviewer Report

Title: "Estimating the total number of phosphoproteins and phosphorylation sites in eukaryotic proteomes."

Version: Original Submission **Date:** 9/7/2016

Reviewer name: **Peter Li**

Reviewer Comments to Author:

This is a well-written manuscript that reports on work estimating the number of phosphoproteins and phosphorylation sites from four eukaryotic organisms: yeast, mouse, human and Arabidopisis. A lot of manual work appears to have gone into this study to mine information about phosphoproteins and phosphorylation sites from over a 1000 articles which the authors responsible for doing this should be commended on. Since I only have a rudimentary knowledge of the biology of phosphoproteins, my comments relate to the reproducibility of the authors' work which is a focus of GigaScience.

Background

Line 73: From the 1000 plus articles that were surveyed, a set of 188 phosphoprotemic datasets were obtained and used for further analysis. The 188 datasets come from the papers listed in 5_published_datasets.txt - can the authors check that this file is referred to in the manuscript like the other supplementary files please?

The 5_published_datasets.txt file lists 185 papers which I found a bit confusing as I wrongly assumed that there would be 188 papers relating to the 188 phosphoprotemic datasets. What appears to have happened is that 3 datasets were obtained from 1 of the yeast publications. A note should be added somewhere to report this detail.

My text editor shows a strange character where a hyphen should be present to separate the starting and ending page numbers in the 5_published_datasets.txt file. Can the authors correct this problem if they see it too please?

It would be good if the 5_published_datasets.txt file contains the Pubmed ID associated with each paper so that it can be cross-referenced with the *_psites.csv files.

Data Description

Line 87 in the Data Description section says that 97, 42, 28 and 20 datasets were retained for human, mouse, Arabidopsis, and budding yeast, respectively. 97, 42, 28 and 20 equals 187 datasets but line 73 says 188 datasets were gathered. Can the authors confirm/correct the number of gathered datasets in

the manuscript?

Methods

Two methods were used to determine number of phosphorylated proteins. The capture-recapture method was done using R with the Rcapture module. It would be good if the authors provided access to the R script to allow readers to see how the capture-recapture method analysis was performed to promote reproducibility for readers.

The curve-fitting on the saturation curve of redundant phosphoproteins/p-sites vs non-redundant phosphoproteins/p-sites is done in MS Excel using the GRG non-linear solving method. I would appreciate if the authors could look into performing this analysis in R to promote reproducibility. However, I am not sure that there is an R module which provides the GRG non-linear solving method for curve fitting - maybe there is information here: http://ebanshi.cc/questions/3525511/generalized-reduced-gradient-grg2-algorithm-in-r

There is a MATLAB calculation (Jaccard distance and similarity) that measures the level of pairwise overlap between pairs of experiments so that highly similar datasets can be excluded in case they inflate the observed saturation of the sampled population. For the sake of completeness, it would be good to include this file and its data as supplementary materials.

I would like to suggest that all of the supplementary data files and scripts be made available in a GitHub repository with readme or documentation files. A link to the GitHub repository can then be referred to in the manuscript.

Discretionary revisions

Check if HTP and LTP need to be listed in abbreviations or written in full when first defined.

I would prefer to see labels for axes being present on the graphs themselves rather described in figure legends.

Methods

Are the methods appropriate to the aims of the study, are they well described, and are necessary controls included? Yes

Conclusions

Are the conclusions adequately supported by the data shown? Choose an item.

Reporting Standards

Does the manuscript adhere to the journal's guidelines on minimum standards of reporting? Yes Choose an item.

Statistics

Are you able to assess all statistics in the manuscript, including the appropriateness of statistical tests used? Yes, and I have assessed the statistics in my report.

Quality of Written English

Please indicate the quality of language in the manuscript: Choose an item.

Declaration of Competing Interests

Please complete a declaration of competing interests, considering the following questions:

- Have you in the past five years received reimbursements, fees, funding, or salary from an organisation that may in any way gain or lose financially from the publication of this manuscript, either now or in the future?
- Do you hold any stocks or shares in an organisation that may in any way gain or lose financially from the publication of this manuscript, either now or in the future?
- Do you hold or are you currently applying for any patents relating to the content of the manuscript?
- Have you received reimbursements, fees, funding, or salary from an organization that holds or has applied for patents relating to the content of the manuscript?
- Do you have any other financial competing interests?
- Do you have any non-financial competing interests in relation to this paper?

If you can answer no to all of the above, write 'I declare that I have no competing interests' below. If your reply is yes to any, please give details below.

I am a friend of the corresponding author, GD Amoutzias. I have previously collaborated and published with SG Oliver's group at the University of Manchester, UK.

I agree to the open peer review policy of the journal. I understand that my name will be included on my report to the authors and, if the manuscript is accepted for publication, my named report including any attachments I upload will be posted on the website along with the authors' responses. I agree for my report to be made available under an Open Access Creative Commons CC-BY license (http://creativecommons.org/licenses/by/4.0/). I understand that any comments which I do not wish to be included in my named report can be included as confidential comments to the editors, which will not be published.

I agree to the open peer review policy of the journal

To further support our reviewers, we have joined with Publons, where you can gain additional credit to further highlight your hard work (see: https://publons.com/journal/530/gigascience). On publication of this paper, your review will be automatically added to Publons, you can then choose whether or not to claim your Publons credit. I understand this statement.

Yes
