# Supplementary material for: Estimating the total number of phosphoproteins and phosphorylation sites in eukaryotic proteomes
Source: Gigascience. 2017 Jan 7;6(2):1–11. doi: 10.1093/gigascience/giw015 (PMC5466708; doi:10.1093/gigascience/giw015)
Supplement: Reviewer_2_Report_(Original_Submission).pdf [file giw015_reviewer_2_report_(original_submission).pdf]

Reviewer Report

Title: "Estimating the total number of phosphoproteins and phosphorylation sites in eukaryotic proteomes."

Version: Original Submission **Date:** 9/25/2016

Reviewer name: **Luca Freschi**

Reviewer Comments to Author:

The authors estimate the number of phosphoproteins and phosphorylation sites in 4 model organisms (Human, Mouse, Arabidopsis and Yeast) and discuss the status of our current knowledge about phosphoproteins and phosphorylation sites.

In my opinion this work represents a valuable contribution to the field of phosphoproteomics. Indeed, it addresses two of the most frequently asked questions in the field: how many phosphoproteins and p-sites are present in model organisms? How many of them do we miss with the current technologies?

I only found two minor issues the authors must fix before submitting the final version of the manuscript:

#1 -- all figures: the axes are almost always not labelled. Although there is a description of each analysis above each graph and the variables are listed in the figure legends, I still feel that it is always a good practice to label the graphs.

#2 -- Figure 2: there are two labels ("CF_best_first", "CF_gest_last") that are never used elsewhere in the manuscript.

I also have one small suggestion to improve the text:

Note for the authors: the line numbers I am referring to are those present in the original Word document.

#1 -- page 4, lines 76-77-- "we have obtained, for the first time, a reliable estimate of their total number for humans and three model eukaryotes.". I would say "we have obtained, for the first time, a reliable estimate of their total number for humans and three other model eukaryotes."

Finally, I found a few typos the authors should correct before submitting the final version of the manuscript:

#1 -- page 1, line 22 -- "high throughput" => "high-throughput".

#2 -- page 6, line 127 -- "Fig, 1B" => "Figure 1B"

#3 -- page 6, line 131 -- "Figure1C" => "Figure 1C"

#4 -- page 7, lines 155-157 -- "In the Table, the most reliable estimates, obtained by incorporating both the HTP and LTP non-redundant datasets is highlighted in bold.". => "In the Table, the most reliable estimates, obtained by incorporating both the HTP and LTP non-redundant datasets are highlighted in bold.".

#5 -- page 10, line 191 -- "Jacknife" => "jackknife"

#6 -- page 11, lines 227-228 -- "lower than those of the human phosphoproteins and p-sites respectively. (See Table 1 for details.)" => "lower than those of the human phosphoproteins and p-sites respectively (See Table 1 for details)."

#7 -- page 12, line 234 -- there are two typos. "14796" => "14,796"; "4930" => "4,930" -- check also the methods section.

#8 -- page 12, lines 236-237 -- "while the estimates on their total number, based on the different methods and data treatments, is depicted in Figure 4B." => "while the estimates of their total number, based on the different methods and data treatments, are depicted in Figure 4B."

#9 -- page 26, line 549 -- "Racpture_HTP_vs_LTP" => "Rcapture_HTP_vs_LTP"

#10 -- page 26, line 554 -- "CF_3x" => "CF_3X"

Methods

Are the methods appropriate to the aims of the study, are they well described, and are necessary controls included? Yes

Conclusions

Are the conclusions adequately supported by the data shown? Yes

Reporting Standards

Does the manuscript adhere to the journal’s guidelines on [minimum standards of reporting?](#) Yes

Statistics

Are you able to assess all statistics in the manuscript, including the appropriateness of statistical tests used? Yes, and I have assessed the statistics in my report.

Quality of Written English

Please indicate the quality of language in the manuscript: Acceptable

Declaration of Competing Interests

Please complete a declaration of competing interests, considering the following questions:

- Have you in the past five years received reimbursements, fees, funding, or salary from an organisation that may in any way gain or lose financially from the publication of this manuscript, either now or in the future?
- Do you hold any stocks or shares in an organisation that may in any way gain or lose financially from the publication of this manuscript, either now or in the future?
- Do you hold or are you currently applying for any patents relating to the content of the manuscript?
- Have you received reimbursements, fees, funding, or salary from an organization that holds or has applied for patents relating to the content of the manuscript?
- Do you have any other financial competing interests?
- Do you have any non-financial competing interests in relation to this paper?

If you can answer no to all of the above, write 'I declare that I have no competing interests' below. If your reply is yes to any, please give details below.

I declare that I have no competing interests

I agree to the open peer review policy of the journal. I understand that my name will be included on my report to the authors and, if the manuscript is accepted for publication, my named report including any attachments I upload will be posted on the website along with the authors' responses. I agree for my report to be made available under an Open Access Creative Commons CC-BY license (http://creativecommons.org/licenses/by/4.0/). I understand that any comments which I do not wish to be included in my named report can be included as confidential comments to the editors, which will not be published.

I agree to the open peer review policy of the journal

To further support our reviewers, we have joined with Publons, where you can gain additional credit to further highlight your hard work (see: https://publons.com/journal/530/gigascience). On publication of this paper, your review will be automatically added to Publons, you can then choose whether or not to claim your Publons credit. I understand this statement.

Yes
