# Supplementary material for: Estimating the total number of phosphoproteins and phosphorylation sites in eukaryotic proteomes
Source: Gigascience. 2017 Jan 7;6(2):1–11. doi: 10.1093/gigascience/giw015 (PMC5466708; doi:10.1093/gigascience/giw015)
Supplement: Reviewer_3_Report_(Original_Submission).pdf [file giw015_reviewer_3_report_(original_submission).pdf]

Reviewer Report

Title: "Estimating the total number of phosphoproteins and phosphorylation sites in eukaryotic proteomes."

Version: Original Submission **Date:** 11/4/2016

Reviewer name: Thierry. Le Bihan

Reviewer Comments to Author:

In this study, the authors proposed a strategy to quantify the relative proportion of the phosphoproteins and phosphopeptide sites present in a few organisms. This is a very interesting question to tackle. The authors proposed two strategies which are: (1) Capture-Recapture and (2) fitting the saturation curve of cumulative redundant vs. cumulative non-redundant phosphoproteins/p-sites. This strategy, if valid, could be applied to other potential protein modifications as well. In addition to quantifying the completeness of the phosphoproteome being identified, the authors provide an exhaustive list of highly confident sites, which can serve as a valuable resource for any future studies and analyses. Therefore, I would like to see this manuscript and the list of highly confident phospho sites published and made available to the scientific community in some way. Yes this study is addressing an interesting question and the collaborative team proposing to address the question seems highly capable; I highlight 2 main/major concerns and few minor points:

1st concern: Need for validating the method. The authors proposed a strategy to answer a question which has an unknown answer, however it is common practice to validate any new strategy on a known dataset. Before testing the method to answer a question with no known answer, the method should be validated on a known proteomics system. Regarding a validation step, why not simply predict the size of a proteome? The genome/proteome size of any model organism is roughly known and this can be used as a validation strategy. Using a small well characterised organism and possibly one organism that is not fully covered by proteomics experiments as well, it could be shown whether the two methods capture-recapture and fitting the saturation curve of cumulative redundant vs. cumulative non-redundant proteins are either close to or far from the predicted number of proteins.

2nd concern: Addressing the bias peptide detection. Most of the phospho-proteomics studies (high or low throughput) have been performed using mainly 3 different enrichment methods, IMAC, titanium dioxide and p-Tyr pull down. While other variations do exist, those are the three most widely used methods. There is a small overlap between the outcome of those three methods which suggests that each of those methods have bias toward certain classes of phosphopeptides (this bias has been frequently reported, a recent review paper such as Fila & Honys, doi: 10.1007/s00726-011-1111-z mention it and this bias is an accepted fact). This could suggest the existence of a pool of phosphopeptides which can't be captured/detected using any of those three phospho-enrichment

methods and will remain a fraction of false negatives. Most likely all detection methods will converge towards a specific value which is the "Detectable phosphoproteome". What is the gap between the Complete phosphoproteome and the Detectable phosphoproteome? This is unknown and I am not sure how that pool of peptides could be tackled; perhaps using a complementary strategy combining absolute protein abundance evaluation with some 32P experiments? Here, I understand that addressing this question with a set of complex experiments is out of the scope of this study, however, the authors should be cautious in their statements being made. For example, in the abstract: "that current high-throughput phosphoproteomics is capable of capturing 70-95% of total phosphoproteins, but only 40-60% of total p-sites." I think the number of phospho-proteins shouldn't be greatly affected, however, the number of peptide p-sites are most likely underestimated. In addition to this enrichment method bias, there is also an underestimation of the number of phosphopeptides due to peptide detectability as any phosphopeptide level determination relies on a good protein coverage by LC-MS. A specific example can be the protein Nanog in which there are some sites which are difficult to detect using standard phospho-proteomics due to their tryptic nature; for example one tryptic peptide stretch is a tryptic peptide of about 12kDa, potentially difficult to detect by LC-MS. In addition, I will digress a bit here; I haven't seen in the supplementary information any protein related to Nanog in mouse for example. Here, either I could have missed it or all of its reported sites didn't fit the high confidence threshold the authors used. Back to this pool of undetectable p-sites, I can imagine those false negatives will also contribute to underestimate the number of possible phosphosites but contrary to the method bias, it should be relatively "easy" for the authors to address this problem. A good strategy to estimate this number would be to determine the global proteome coverage, not in terms of the proteins identified but rather in terms of peptides covered by LC-MS. If we take yeast for example ; what is the overall coverage of the yeast peptidome itself? Assuming it is 80% (using a similar ID confidence used for the p-site), we can assume that the estimated phosphoproteome in this study should be 80% of the real value as the 20% refers to those peptides which are too short, too long, too hydrophobic, etc. to be detected. Assuming as well, a uniform distribution of the p-site across the proteome. In conclusion, 2 different experimental factors contribute to reduce p-site detection:

1) Method Bias

2) The proportion of peptides that can be detected by mass spectrometry.

Suggested approach to tackle this problem: 1) Tone down any mention to "exact" quantitative phosphoproteome coverage by acknowledging the existence of a pool of non-detectable phosphopeptides (I would have coined that the "Dark-proteome" but the term has been already used for another reason). 2) Evaluate a global proteome coverage (using a similar stringency criterion to the one you use for your p-sites), to estimate the fraction of detectable proteome.

3rd concern (less major than the 2 previous one): Regarding the nature of the supplementary information. The Supplementary information tables are not too intuitive in their current format to be of use, unless you are an expert in database/bioinformatics.

In order to highlight the difficulty of using the data in that format, it took me some time to look at 3 sites and discover some key errors. There is an apparent systematic shift in the numbering of the amino acid phosphorylation sites. I only looked at 3 examples in yeast and none of them are correct:

YDR033W_288 is wrong and should be YDR033W_289

YDL051W_18 is wrong and should be YDL051W_19

YJL026W_14 is wrong and should be YJL026W_15

I haven't checked the other species, but I strongly encourage the authors to correct this possible systematic error. If widespread, I would also encourage a "sanity check" such as randomly picking 500-1000 sites from their supplementary information and checking their correctness. This should also be added in the Methods section. The authors should double-check the other species and address this shift if it is has happened across all datasets being reported. I would consider the supplementary data publishable only after fixing this problem and validating them. I would have preferred that the following information be provided as well: the list of peptides themselves (tryptic or a 23 aa window centred on the p-site), their associated protein IDs and any form of confidence score (peptide identification and phosphosite assignment). While this last request is not absolutely necessary, it will contribute greatly to the data accessibility and usefulness to the wider scientific community.

Minor points:

In data description: please provide the version/date of the different databases used.

Figure 1,2,3 and 4, A and C please define the nature of your axis (i.e. an axis title).

Please ensure that all your acronyms are properly introduced at their first mention (HTP) line 105, LTP line 98

Line 347 :''phosphoproteins/p-sites (y-axis) identified..'' Which y-axis figure are you referring too?. Idem at line 348, 353, etc… please refer to a SPECIFIC figure as it is not easy to guess here. If found elsewhere in the text, please ensure you present clearly the figure you are referring to.

Methods

Are the methods appropriate to the aims of the study, are they well described, and are necessary controls included? No

Conclusions

Are the conclusions adequately supported by the data shown? No

Reporting Standards

Does the manuscript adhere to the journal's guidelines on minimum standards of reporting? Yes

Statistics

Are you able to assess all statistics in the manuscript, including the appropriateness of statistical tests used? No, and I do not feel adequately qualified to assess the statistics.

Quality of Written English

Please indicate the quality of language in the manuscript: Choose an item.

Declaration of Competing Interests

Please complete a declaration of competing interests, considering the following questions:

- Have you in the past five years received reimbursements, fees, funding, or salary from an organisation that may in any way gain or lose financially from the publication of this manuscript, either now or in the future?
- Do you hold any stocks or shares in an organisation that may in any way gain or lose financially from the publication of this manuscript, either now or in the future?
- Do you hold or are you currently applying for any patents relating to the content of the manuscript?
- Have you received reimbursements, fees, funding, or salary from an organization that holds or has applied for patents relating to the content of the manuscript?
- Do you have any other financial competing interests?
- Do you have any non-financial competing interests in relation to this paper?

If you can answer no to all of the above, write 'I declare that I have no competing interests' below. If your reply is yes to any, please give details below.

I declare that I have no competing interests

I agree to the open peer review policy of the journal. I understand that my name will be included on my report to the authors and, if the manuscript is accepted for publication, my named report including any attachments I upload will be posted on the website along with the authors' responses. I agree for my report to be made available under an Open Access Creative Commons CC-BY license (http://creativecommons.org/licenses/by/4.0/). I understand that any comments which I do not wish to be included in my named report can be included as confidential comments to the editors, which will not be published.

I agree to the open peer review policy of the journal

To further support our reviewers, we have joined with Publons, where you can gain additional credit to further highlight your hard work (see: https://publons.com/journal/530/gigascience). On publication of

this paper, your review will be automatically added to Publons, you can then choose whether or not to claim your Publons credit. I understand this statement.

Yes
